# Bioinformatics tools and data resources for assay development of fluid protein biomarkers

**DOI:** 10.1186/s40364-022-00425-w

**Published:** 2022-11-15

**Authors:** Katharina Waury, Eline A. J. Willemse, Eugeen Vanmechelen, Henrik Zetterberg, Charlotte E. Teunissen, Sanne Abeln

**Affiliations:** 1 IBIVU - Center for Integrative Bioinformatics, Department of Computer Science, Vrije Universiteit Amsterdam, Amsterdam, The Netherlands; 2 Departments of Biomedicine and Neurology, MS Center and Research Center for Clinical Neuroimmunology and Neuroscience Basel (RC2NB), University Hospital Basel, Basel, Switzerland; 3 Department of Clinical Chemistry, Amsterdam Neuroscience, VU University Medical Center, Amsterdam, The Netherlands; 4 ADx NeuroSciences, Ghent, Belgium; 5 Department of Psychiatry and Neurochemistry, Institute of Neuroscience and Physiology, Sahlgrenska Academy at the University of Gothenburg, Mölndal, Sweden; 6 Clinical Neurochemistry Laboratory, Sahlgrenska University Hospital, Mölndal, Sweden; 7 Department of Neurodegenerative Disease, UCL Institute of Neurology, London, UK; 8 Hong Kong Center for Neurodegenerative Diseases, Hong Kong, China; 9 CWI, Amsterdam, The Netherlands

**Keywords:** Biomarker, Immunoassay, Antibody, Dementia, Bioinformatics

## Abstract

Fluid protein biomarkers are important tools in clinical research and health care to support diagnosis and to monitor patients. Especially within the field of dementia, novel biomarkers could address the current challenges of providing an early diagnosis and of selecting trial participants. While the great potential of fluid biomarkers is recognized, their implementation in routine clinical use has been slow. One major obstacle is the often unsuccessful translation of biomarker candidates from explorative high-throughput techniques to sensitive antibody-based immunoassays. In this review, we propose the incorporation of bioinformatics into the workflow of novel immunoassay development to overcome this bottleneck and thus facilitate the development of novel biomarkers towards clinical laboratory practice. Due to the rapid progress within the field of bioinformatics many freely available and easy-to-use tools and data resources exist which can aid the researcher at various stages. Current prediction methods and databases can support the selection of suitable biomarker candidates, as well as the choice of appropriate commercial affinity reagents. Additionally, we examine methods that can determine or predict the epitope - an antibody’s binding region on its antigen - and can help to make an informed choice on the immunogenic peptide used for novel antibody production. Selected use cases for biomarker candidates help illustrate the application and interpretation of the introduced tools.

**Supplementary Information:**

The online version contains supplementary material available at 10.1186/s40364-022-00425-w.

## Introduction

Biomarkers comprise biological measurements that can give an indication about a person’s medical state, disease progression or response to intervention [1]. Thus, biomarkers can be critical for prognosis, diagnosis, disease sub-typing and monitoring of disease advancement or treatment response [1]. Fluid biomarkers specifically are biomolecules that can be detected and quantified in one of the bodily fluids, such as blood plasma or cerebrospinal fluid (CSF). Their inexpensive and often minimally invasive sample collection renders fluid biomarkers suitable for a broad clinical use and is therefore the focus of many medical research fields [2, 3]. Biological fluids often provide the only viable option to examine the protein profile of the tissue of interest [4]. For instance, because of its close proximity to the brain, CSF is especially pertinent for neurological disorders such as dementias [4]. However, with the ongoing advancement of ultra-sensitive measurement technology the translation of CSF- to blood-based biomarkers is also actively pursued [2, 5]. The potential of fluid protein biomarkers is immense, especially to tackle current major challenges within the dementia field [6]. Novel and robust biomarkers are needed to allow an early and correct diagnosis, as identification based on clinical manifestation alone is still a challenging and lengthy process and often inaccurate, since dementia can develop due to multiple causes [7–9]. The complexity of dementia pathology suggest that a combination of protein biomarkers may be necessary for accurate conclusions and thus the use of biomarker panels is increasingly explored [10, 11]. Additionally, it has become clear that the pathological processes in neurodegenerative diseases may start decades before clinical symptoms manifests. Therefore, in clinical trials that target the early stages of the diseases, fluid biomarkers are required to enable an improved pathology-based participant selection. Moreover, biomarkers allow the monitoring of adverse events and endpoints for trials [12]; this is key to increase the success of dementia drug trials. Despite the promise of fluid biomarkers, the implementation in clinical use has been slow and their potential is still largely untapped [13–15]. As biological fluids are complex matrices, reliable biomarker detection is only possible with highly sensitive and specific assays.

In this review we will consider how the development of fluid protein biomarker assay methods can be supported, using bioinformatics tools and data resources. While we will focus on the domain of dementia biomarker development, the recommendations given here can be used in the setting of any protein biomarker or biomarker panel.

### The development of novel biomarkers

Novel biomarker development is a long and multidisciplinary process that consists of biomarker discovery, qualification, verification and clinical validation [16, 17].

Biomarker discovery has the aim to identify novel proteins that are most suitable to differentiate between two states of interest (e.g., disease vs. non-disease) by means of their expression levels [18]. There are two principal approaches to biomarker discovery. One depends on knowledge of the underlying disease process and targeted selection and development of biomarker candidates. The other, which is relevant to this review, is explorative. An explorative approach typically uses hypothesis-free experimental techniques allowing the simultaneous detection of many proteins to increase the success of candidate identification. While untargeted mass spectrometry is the customary method of choice for biomarker discovery [19], novel multiplexed antibody- or aptamer-based proteomics methods are increasingly utilized as well [20–22]. We outline the advantages and disadvantages of these three approaches in Table 1 [20]. Because of their complementary nature, integration of these methods has recently been suggested [23, 24].

**Fig. 1:**
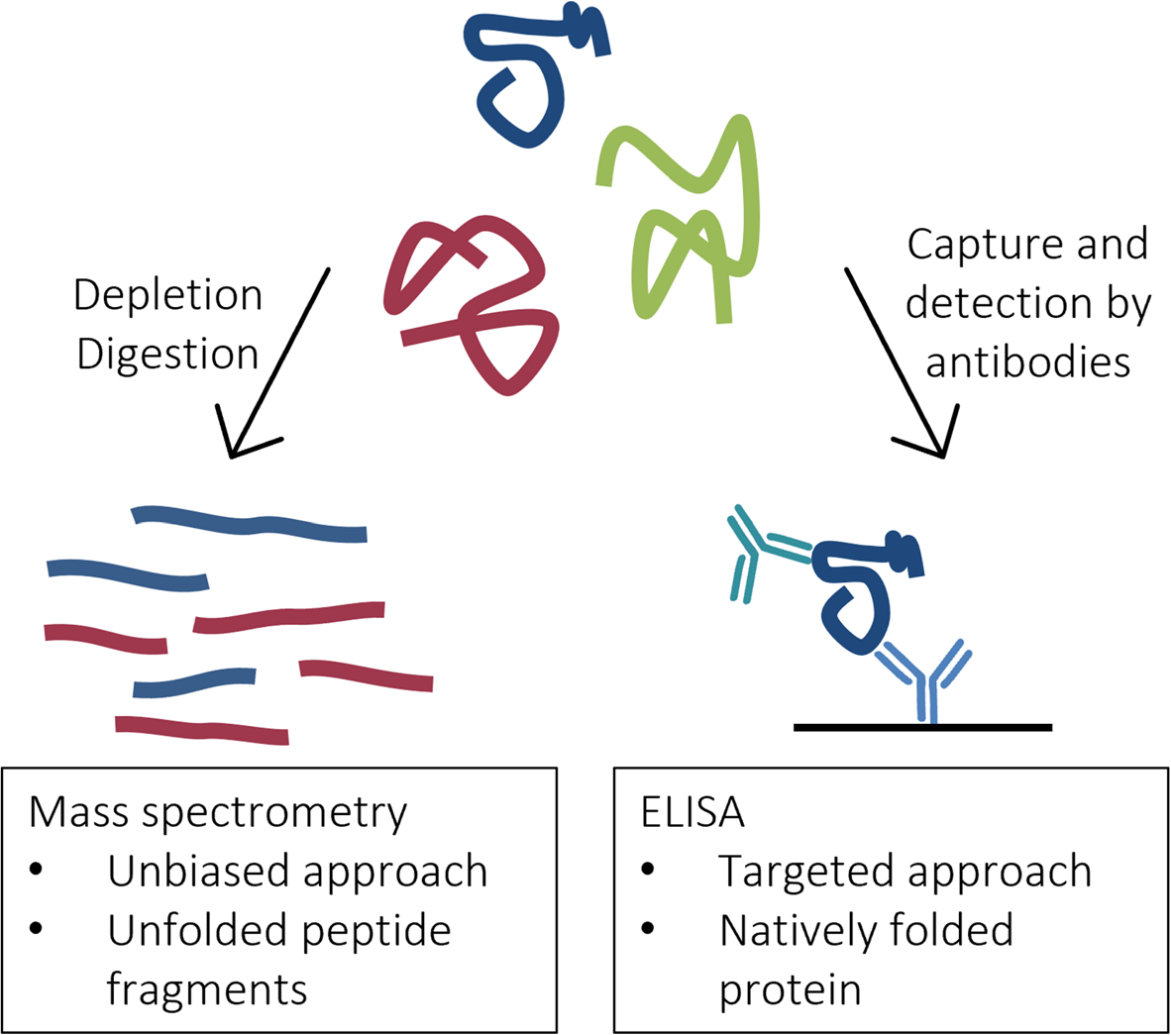
Difference in sample preparation between MS and immunoassay technologies. MS is an unbiased detection method; the sample fluid is depleted of highly abundant proteins and proteolytically digested before measurement. Immunoassays allows targeted protein detection by antibodies; the protein target is in its native fold during measurement

Because of its widespread prominence and use, we focus on MS as a biomarker discovery tool in depth hereinafter. The relative protein quantification using MS is facilitated through the ionization of the present biomolecules, followed by their separation and detection based on their mass-to-charge ratio. Importantly, preceding sample preparation usually involves the depletion of highly abundant proteins and protein digestion by a protease [25] (Fig. 1). Thus, instead of full-length proteins MS detects peptide fragments which need to be mapped to the corresponding protein afterwards [19]. As a result of the digestion step any protein folding and interaction is eliminated from the MS samples before their detection.

**Table 1:** Commonly used technologies for biomarker discovery. Multiple approaches exist that allow multiplexed protein measurements in human body fluid samples. While mass spectrometry is still the customary method, novel affinity-based proteomics methods are also increasingly utilized

Technology	Advantages	Disadvantages	Availability	Relevant references
Untargeted mass spectrometry	Unbiased approach, widely established and used in laboratories, proteoform-specific information	False positive rate, limited sensitivity and dynamic range	No restriction	[26]
Proximity extension assay	High dynamic range, sensitivity and specificity	Not available for all proteins, no assay adaptation	>3000 proteins	[21]
Aptamer-based proteomics	High dynamic range, sensitivity and specificity	Not available for all proteins, no assay adaptation	>7000 proteins	[22]

One of the major drawback of MS is the low achievable sample throughput because of the intensive sample preparation and the high associated costs of this technology [27]. The limited number of samples can lead to a high false positive rate for biomarker candidates; consequently, a following verification using an increased number of samples is essential [28]. While the qualification and verification of a continuously funneled set of biomarker candidates might still be performed by targeted MS technologies, clinically used biomarker assays eventually require a more widely accessible, cost-effective and high sample throughput technology that still offers high sensitivity [13]. The most established method for validation and routine clinical use is the antibody-based immunoassay, most commonly in the format of an enzyme-linked immunosorbent assay (ELISA) [13, 17]. Note that many alternative immunoassay technologies with higher sensitivity and associated costs exist that have been summarized elsewhere and are not further considered here [29].

ELISA is a targeted immunodetection approach that is customarily implemented as a “sandwich” assay. These immunoassays allow the detection of protein biomarkers by capturing and immobilizing the protein target with a first *capture* antibody, while producing a read-out signal through the second *detection* antibody binding to the target [30] (Fig. 1). The strength of the signal correlates with the amount of the target bound, and thus allows the absolute quantification of the protein in the sample [30]. The application of antibodies allows high flexibility and sensitivity, two of the main advantages of immunoassays. Antibodies can be raised against virtually any kind of biomolecule and will detect their target at extremely low concentration even in complex samples such as plasma [31]. The identification of a favorable pair of a capture and a detection antibody for a specific biological matrix and concentration range is a crucial part of the development of novel biomarker assays. If the assay has been validated and optimized, its performance can be validated in a large patient cohort, before being commercially pursued and brought to the market [18].

Owing to their prominence and prevalent use, here we described an MS-to-ELISA-centered biomarker development pipeline. Note that the arguments made in the following are applicable to any workflows with the aim of establishing clinical immunoassays based on explorative biomarker studies such as mass spectrometry, proximity extension assays or aptamer-based proteomics [19, 21, 22].

### The cross-technology translation gap

Methods for biomarker discovery and clinical validation exhibit benefits and weaknesses that make them suitable for one step but inadequate for the other [27]. They should thus be considered complementary. While there is an ongoing effort to hybridize and improve biomarker detection methods [21, 32, 33], the translation of results from exploratory to targeted approaches is still an important process to arrive at biomarkers for clinical practice. Herein lies one of the major challenges of the current pipeline: the cross-technology translation gap [13]. Biomarker discovery, e.g., the analysis of samples by MS, often leads to the identification of many proteins showing differing levels and thereby to a lengthy list of biomarker candidates. But those measurement differences in protein levels can often not be replicated on the immunoassay platform, thereby halting the development pipeline. This issue might often be caused by the differences in sample preparation and protein detection between the technologies, e.g., between MS and ELISA (Fig. 1).

Several other difficulties and gaps arise in the pipeline of biomarker development which have been the subject of previous reviews [13, 18, 19, 34] and are not considered here further. Instead, we concentrate on the bottleneck of cross-technology translation and aim to present the specific stages at which bioinformatics tools might be incorporated into novel assay design to bridge this gap. Due to the rapid progress within the field of bioinformatics, many resources are nowadays available that can aid this challenging process. To the best of our knowledge, this work is the first to provide recommendations on how to apply these tools specifically for biomarker assay development to identify and overcome potential obstacles. Specific examples of current prediction methods and databases are provided with the hope that this review offers a resourceful starting point for the interested researcher.

## Bioinformatics workflow for biomarker assay development

**Fig. 2:**
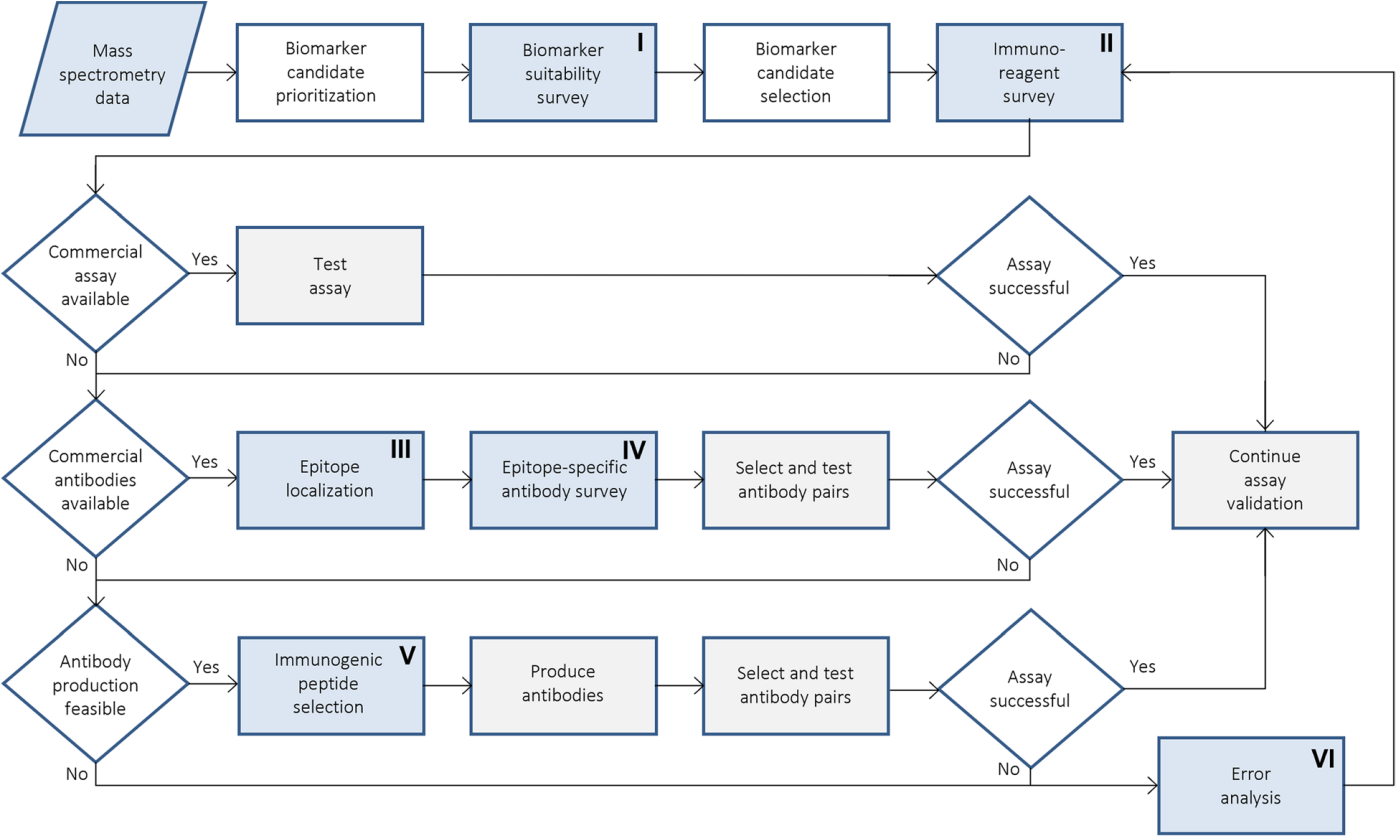
Workflow for novel immunoassay development. A flowchart of typical steps to follow for optimal assay development of biomarker candidates identified by explorative approaches. I-VI: boxes highlighted in blue indicate stages at which bioinformatics might be incorporated. The associated areas of interest are given in section 2. For detailed information on specific methods and databases associated with each named step in the workflow see section 3 and Table 2

Based on the typical approach for the immunoassay development of a novel biomarker target, we wish to highlight several stages at which bioinformatics tools could enhance the process and increase the chances of successful assay design. A proposed workflow of novel immunoassay development is shown in Fig. 2 with steps to incorporate bioinformatics highlighted. This workflow does not include detailed steps for the actual validation experiments; instead, we focus on the preceding decisions regarding biomarker candidate, antibody and immunogenic peptide selection. In this section, we aim to illustrate the relevance and benefit of those steps within the complete workflow and to define which properties are of interest at which point. The subsequent section will contain the detailed description of bioinformatics resources considered to be helpful, and specifically how those tools can be utilized to identify possible obstacles and solve arising difficulties. Furthermore, the areas of interest highlighted in bold in this section can be found in Table 2 to find the associated tools and resources; immunoreagent databases were separately collected in Table 3.

### Biomarker suitability survey

Following the discovery of biomarker candidates by exploratory proteomics studies or other approaches such as genetic studies or biological pathway analyses [19, 35, 36], the first step of assay development should be a thorough and critical evaluation of those proteins. A selection of a limited number of proteins is often necessary, as the efforts and costs associated with assay validation are too great for a multitude of proteins [17]. It is therefore important to be able to single out the most promising candidates. The difference in protein levels between groups is still the principal consideration for the prioritization of biomarker candidates and different strategies to select biomarker candidates from proteomics results have been compared elsewhere [37]. However, this selection can be augmented by additional information about the proteins’ suitability as biomarkers and as immunoassay targets. Attaining more knowledge about **biological context**, **protein origin and location**, **structural protein features**, and **proteoform complexity** is vital to reveal obvious reasons to include or exclude biomarker candidates.

Even if a prioritization of biomarker candidates is not required, e.g., if only a single protein biomarker will be investigated, its characterization can still be advantageous.

### Immunoreagent survey

Available immunoreagents should be surveyed for the chosen protein targets. Commercial immunoassay kits offer the advantage of an antibody pair determined by the manufacturer and are often preferred. However, if the assay is not performing successfully or no kit is available, purchase and validation of commercial, or generation of novel antibodies is needed. It is advantageous to browse available **immunoreagent databases** to consider which antibodies will fit the researcher’s requirements, e.g., regarding validation experiments, specificity and modifications. Experimental validation of the chosen antibodies at this point is recommended if no sufficient data is provided by the supplier [38].

### Epitope localization

In the case that a novel antibody pairing needs to be established for an assay, the choice and combination of tested antibodies is often done arbitrarily and based on a trial-and-error approach [39]. This is especially difficult if no trustworthy validation data for the antibodies is available yet. The application of bioinformatics enables to rationalize that process to a greater extend. One way bioinformatics can support assay development at this stage is through the localization of the distinct area on the protein target, also referred to as the antigen, that the antibody will bind to. This area is called the **epitope** and can either be a single stretch of amino acids (i.e., a linear epitope) or a patch of amino acids brought together closely by the fold of the protein (i.e., a conformational epitope) [40].

While manufacturers often disclose the immunogen, i.e., the protein or peptide fragment against which the antibody was raised, this information is not necessarily enlightening. For methods such as ELISA, that are based on the recognition of the native protein, antibodies raised against the full-length natively folded protein are preferred [41, 42]. Thus, as the immunogen contains the majority of the protein sequence, many areas on the protein surface might constitute the epitope. On the other hand, it might also occur that only antibodies raised against peptide fragments are available as these comprise most commercially available immunoreagents [43]. While exact epitopes can be experimentally identified by epitope mapping, the process is effortful and costly [44]. Bioinformatics provides an easy and fast approach to approximate and study potential epitope locations which might then be evaluated regarding their suitability for the immunoassay. However, the use of computational tools is still limited and cannot be seen as a substitute of experimental epitope determination.

### Epitope-specific antibody survey

Approximating the epitopes of considered antibodies in more detail allows to investigate those areas more thoroughly. Bioinformatics tools can support to identify potential obstacles of antigen-antibody-interaction which would hinder the protein detection in the assay. Specifically investigating the epitope-containing region of the protein target, it can be helpful to examine **structural protein features**, **proteoform complexity** and **protein interactions**. Moreover, the **epitope** regions should be investigated regarding their specificity and overlap with each other. Thus, bioinformatics resources can facilitate the selection of an antibody pair with favorable epitopes that are distant from each other. Less combinations of antibodies might need to be tested to identify a suitable pairing. Additionally to computational approaches, experimental study of antibody characteristics might also be advantageous. Screening for antibody affinity can usually be added early in the selection process; an example is the use of off-rate screening [45].

### Immunogenic peptide selection

If no commercial antibodies exist or perform acceptably, production of novel antibodies might be considered for a strong biomarker candidate; it is however a long and costly endeavor [46]. Antibody production may be undertaken by research groups themselves or can be outsourced to specialized companies. Here, several decisions regarding the antibody specifics have to be made at the beginning regarding clonality (monoclonal vs. polyclonal) and immunogen (full-length protein vs. immunogenic peptide). The use of immunogenic peptides can be advantageous and cost-saving when the full-length protein antigen is difficult to purify and handle [43]. Working with a peptide can give a higher control over the antibody recognition site, but at the same time carries the risk of choosing an epitope shared by other proteins, thus reducing specificity. Therefore, identifying the regions that would be most and least suitable as an epitope in the native protein, can support the production of well functioning antibodies. Similarly to antibody pair selection, it is important to consider **structural protein features**, **proteoform complexity**, **protein interactions**, and **epitopes** to facilitate the production of adequate antibodies.

### Error analysis

If the assay development failed, an option is to revisit the list of potential biomarker candidates to make a novel selection. Beforehand, an error analysis should be performed on previously tested assays by applying any omitted bioinformatics tools regarding **biological context**, **protein origin and location**, **structural protein features**, **proteoform complexity**, **protein interactions** and **epitope** to understand the potential reasons of failure. An error analysis could reveal the unsuitability of the target protein or the chosen antibody pairing. This in turn might lead to an adapted experiment set-up that produces the desired results.

## Bioinformatics tools for biomarker assay development

In the previous section various areas of interest during assay development were highlighted. Here, we wish to detail for each area which specific properties can be investigated, how bioinformatics can be utilized for these tasks at hand, and to introduce specific tools. Only freely available, easy-to-use and web-based methods and databases are considered in this review. We provide at least one state-of-the-art example while also considering reliability. Note that the pace at which new tools are released differs strongly between research fields. This is by no means an exhaustive or complete list. Where possible, more expansive literature on a certain bioinformatics topic is referenced. A summary of all mentioned resources as well as a reference to their associated steps within the workflow of novel immunoassay development (Fig. 2) can be found in Table 2 to allow easy cross-referencing between the workflow (section 2) and the tools (section 3). Additionally, use cases for three Alzheimer’s Disease (AD) biomarker candidates in the following section provide an illustrative application for many of the introduced tools.

**Table 2:** Bioinformatics resources for immunoassay development. All described bioinformatics tools are categorized by the area of interest and further by the specific property that can be examined. Information is provided on the resource type, the required input, and the associated step(s) of the bioinformatics workflow (see Fig. 2). Abbreviations: *AB-Seq* - antibody sequence in FASTA format, *C* - calculation, *DA* - database of annotations, *DP* - database of predictions, *N* - protein name, *P* - prediction method, *PDB* - PDB ID, *Seq* - protein sequence in FASTA format, *Str* - protein structure in PDB format, *UP* - UniProt ID, *VS* - visualization. Workflow steps: *I* - Biomarker suitability survey, *III* - Epitope localization, *IV* - Epitope-specific antibody survey, *V* - Immunogenic peptide selection, *VI* - Error analysis. *These tools can only be utilized if a PDB entry, i.e., a protein structure, for the target exists. ** EpiPred requires the antibody structure. However, the antibody sequence is sufficient if homology modeling is used to derive a structure model from it

Area of interest	Specific property	Bioinformatic tool	Label	Input	Step	References
Biological context	Protein function	UniProtKB	DA	N, UP	I, VI	[47]
		QuickGO	DA	N, UP		[48]
		NetGO 2.0	P	Seq		[49]
	Interaction partners	STRING	DA, DP	N, UP, Seq		[50]
	Disease involvement	OMIM	DA	N		[51]
		DisGeNET	DA	N, UP		[52]
Protein origin and location	Tissue-specific expression	Expression Atlas	DA	N, UP	I, VI	[53]
		Human Protein Atlas	DA	N, UP		[54]
		Human Body Fluid Proteome	DA	N, UP, Seq		[55]
	Subcellular location	UniProtKB	DA	N, UP		[47]
		DeepLoc-2.0	P	Seq		[56]
	Extracellular vesicle localization	Vesiclepedia	DA	N		[57]
Protein structure	Molecular weight	Compute pI/MW	C	UP, Seq	I, IV, V, VI	[58]
	Solved protein structure	Protein Data Bank*	DA	N, UP, Seq, PDB		[59, 60]
	Homology modeling	SWISS-MODEL (Repository)	P, DP	UP, Seq		[61, 62]
	Predicted protein structure	AlphaFold Protein Structure Database	DP	N, UP		[63, 64]
	Structure viewer	Mol* (Mol star)*	VS	PDB, Str		[65]
	Structural protein features	PredictProtein	P	Seq		[66]
		NetSurfP-2.0	P	Seq		[67]
		DescribePROT	DP	UP, Seq		[68]
	Disorder	DisProt	DA	N, UP		[69]
		MobiDB	DA, DP	N, UP		[70]
		IUPred3	P	UP, Seq		[71]
Proteoform complexity	Isoforms and cleavage products	UniProtKB	DA	N, UP	I, IV, V, VI	[47]
	Post-translational modifications	PhosphoSitePlus	DA	N, UP		[72]
		iPTMnet	DA	N, UP		[73]
		MusiteDeep	P	Seq		[74]
Protein interactions	Interacting residues	PredictProtein	P	Seq	IV, V, VI	[66]
		DescribePROT	DP	UP, Seq		[68]
		HybridPBRpred	P	Seq		[75]
		ANCHOR2	P	UP, Seq		[76]
		InterPro	DA	N, UP, Seq		[77]
		MobiDB	DA, DP	N, UP		[70]
	Aggregation	Aggrescan3D 2.0*	P	PDB, Str		[78]
		PASTA 2.0	P	Seq		[79]
		AmyPro	DA	N, UP, Seq		[80]
Epitopes	Epitope prediction	BepiPred-2.0	P	Seq	III, IV, V,VI	[81]
		SeRenDIP-CE	P	Seq		[82]
		ElliPro*	P	PDB, Str		[83]
		epitope3D*	P	PDB, Str		[84]
	Antibody-specific epitope prediction	EpiPred*	P	PDB, Str, AB-Seq**		[85, 86]
	Known epitopes	IEDB	DA	N, UP		[87]
		SAbDab*	DA	N, PDB		[88]
	Epitope specificity	BLAST	C	Seq		[89]

### Biomarker candidate ID

As an initial step, every potential biomarker’s associated UniProt entry should be identified. The UniProt Knowledgebase [47] provides an expansive collection of protein entries that contain their sequence, existing annotations and cross-references to other databases and thereby offers an immense collection of valuable knowledge in itself. UniProt also contains the UniProt ID (or accession number) and canonical amino acid sequence that are often required as input for other bioinformatics tools and databases and enable the unique and stable identification of each protein.

### Biological context

Protein function can elucidate if alteration to the protein expression could be associated with the pathology of interest. Function can be well characterized by the Gene Ontology (GO) annotations. GO is a defined and consistent vocabulary that assigns to every protein associated terms of three major categories: biological processes, molecular functions, and cellular components [90, 91]. A protein’s GO terms can be viewed in UniProt entries or more thoroughly via specific online tools, e.g., QuickGO [48]. Prediction of protein function is available for proteins that lack (a complete) functional annotation albeit it is a difficult prediction problem: NetGO 2.0 is currently the state-of-the-art predictor and only requires the protein sequence as input on its webserver [49].

Knowledge about disease associated interaction partners can also increase the confidence in a biomarker candidate. STRING is a protein-protein-interaction database collecting information from a vast number of sources such as text mining, databases, experimental evidence and computational predictions [50]. The interaction network for a given protein is presented in a graph-based manner and analysis of functional enrichment within the network is included as well.

If involvement in disease can be identified, it noticeably increases the confidence in a candidate’s capacity as a biomarker. Furthermore, it might be important to establish the protein’s “specificity” as a biomarker for the intended use. One example of an “unspecific” diagnostic biomarker is neurofilament light; it reliably indicates axonal damage and is thus considered a cross-disease biomarker for axonal damage in neurological disorders [92]. This however limits its suitability for the differential diagnosis of a specific brain pathology as the protein shows increased levels in various conditions. Curated disease-association databases give insight if the protein of interest has been implicated in a pathological state. The Online Mendelian Inheritance in Man (OMIM) is a database focused on inheritable diseases and provides a comprehensive overview of available literature and evidence for its gene-phenotype relationships [51]. The DisGeNET database integrates information from multiple sources about human gene-disease associations and ranks the associations by relevance [52].

### Protein origin and location

Especially for fluid biomarkers, it is worthwhile to determine a protein’s likely origin by examining its tissue-specific expression. Body fluids can contain proteins secreted from various organs and tissues; the detection in a fluid is therefore not providing much certainty yet on a protein’s origin. For instance, only approximately 20% of proteins found in CSF are brain-derived [93]. If a CSF-detected protein is predominantly expressed in the brain, it would strongly increases its promise as a biomarker for neuropathologies.

Two major protein expression resources are the Expression Atlas and the Human Protein Atlas (HPA). Both atlases provide trustworthy data on the tissues in which a protein has been detected. The Expression Atlas is a curated collection of gene expression results providing information on the tissues in which a protein is expressed and how it changes during disease [53]. Similarly, the HPA intents to map the entire human proteome to their respective tissues and organs and offers in addition to a comprehensive Tissue Atlas, also more specialized collections, e.g., the Blood Atlas and the Brain Atlas [54]. The HPA also provides knowledge about the human secretome which is of high interest for biomarker candidates, especially if pathological processes are linked to the secretion of these proteins [94]. A further resource especially relevant for fluid biomarker research is the Human Body Fluid Proteome [55]. It is a collection of 17 types of body fluid proteomes (including blood and CSF) which offers a confidence score for each human protein to be detected in a specific fluid based on previously published studies.

The protein’s subcellular localization can show its potential as a fluid biomarker as its presence in the extracellular region might corroborate its subsequent presence in body fluids. Furthermore, location can provide additional context on the protein’s function [95]. UniProt will contain annotations about the associated cell organelles. As often these annotations will not be complete, subcellular localization predictors are available for further exploration. For instance, DeepLoc-2.0 is a novel method able to predict in which compartment(s) a protein is likely localized [56].

**Fig. 3:**
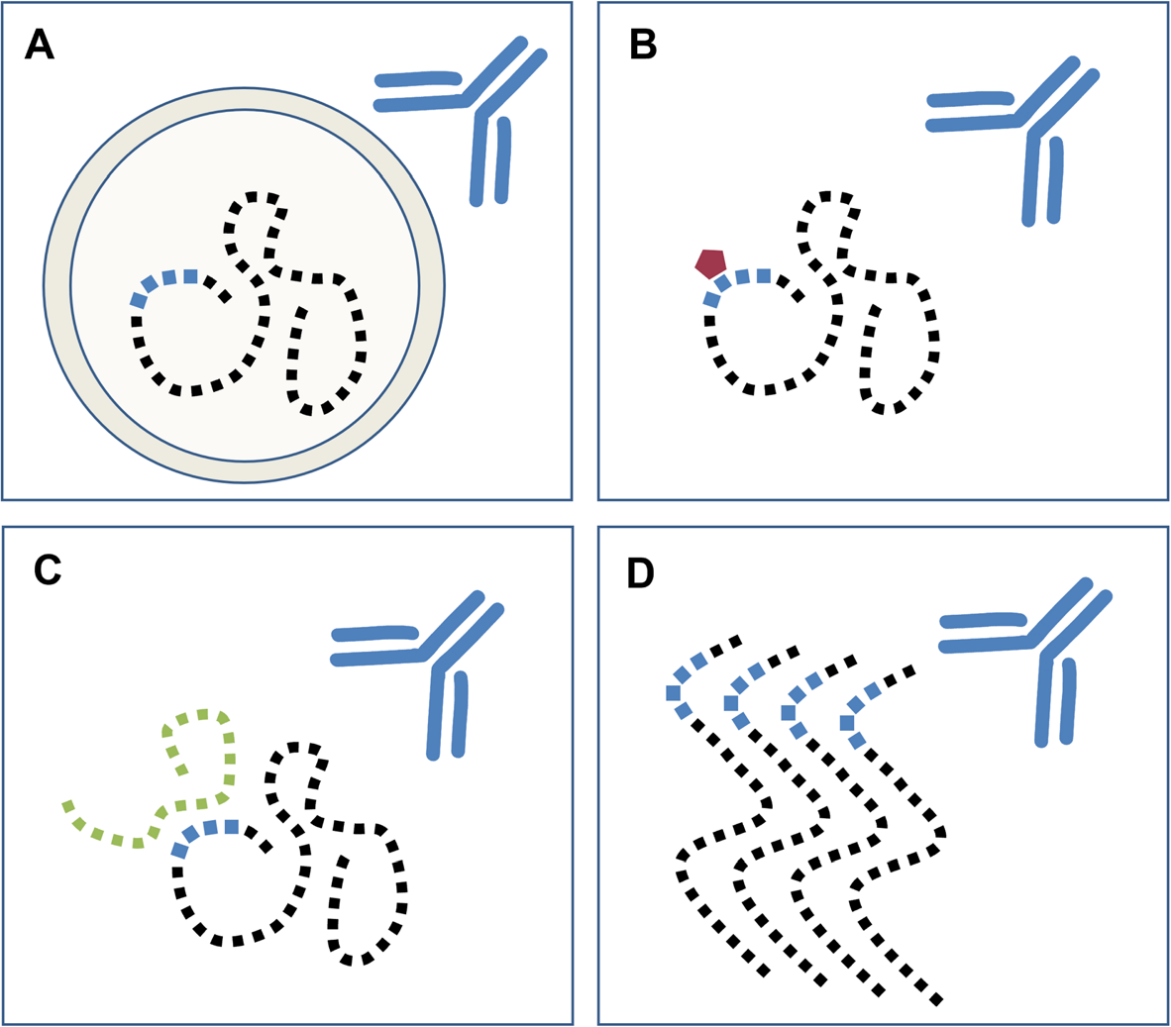
Potential matrix-dependent obstacles of successful antibody binding to fluid protein biomarkers. Several circumstances can arise within an immunoassay that hide the epitope (blue) from its corresponding antibody. (A) Fluid biomarkers that are located inside EVs are not accessible for antibodies. (B) A PTM (red) that lies on or close to the epitope might mask it from the antibody. (C) If the interface of a protein interaction with another present molecule (green) is close or on the epitope, the antibody cannot recognize its binding region. (D) If a protein is accumulating, its altered structure might hide the epitope within the aggregate

A more specific, but relevant, circumstance is the presence of the biomarker in an extracellular vesicle (EV) within the body fluid (Fig. 3A). EVs are secreted by virtually all cell types and are thought to facilitate cell-to-cell communication [96]. Their cargo proteins are often considered strong biomarker candidates as they provide insight into the state of the originating cells and have been detected in many fluids [97]. Moreover, they have been implicated in the propagation of pathologies such as cancer and neurodegenerative diseases [98, 99]. Interestingly, brain-derived EVs are able to cross the blood-brain-barrier and have previously been isolated from CSF and plasma [98, 100]. However, the protein cargo can only be accessed by assay antibodies if those vesicles are isolated and disrupted beforehand [101, 102]. As appropriate steps, e.g., ultracentrifugation, are not included in the ELISA workflow, one should be aware of a biomarker’s association with EVs. The EV cargo database Vesiclepedia [57] asserts if a protein of interest has been found in those vesicles in previous studies.

### Structural protein features

**Fig. 4:**
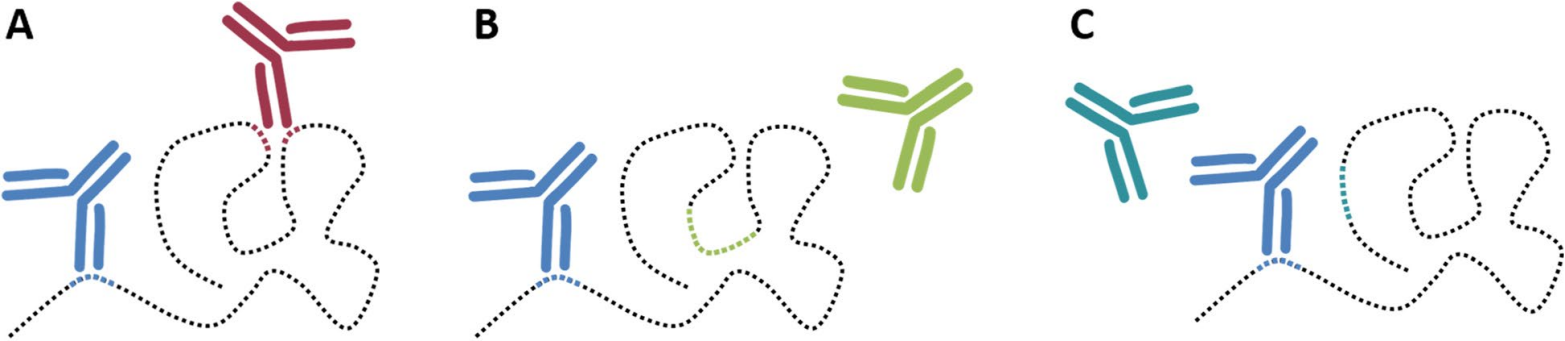
Structural considerations of accessible and inaccessible epitopes in a sandwich immunoassay. (A) Two antibodies can simultaneously bind to the same antigen by interacting with their respective linear (blue) or conformational (red) epitope at the surface of the protein structure. (B) The antibody cannot recognize its epitope (green) as it is buried inside the protein structure, binding to the antigen is unsuccessful. (C) Two epitopes are close together within the native protein fold and one antibody cannot bind its epitope (teal) as it is blocked by the already bound antibody (blue); simultaneous binding to the antigen is unsuccessful

Structural protein features might be utilized to explore the protein’s suitability as an immunoassay target considering the amount of accessible surface area (ASA) that antibodies can bind to. Information on protein structure is also vital to determine the localization of epitopes or potential immunogenic peptides within the full protein. An epitope needs to lie at the surface of the protein target to allow antibody binding (Fig. 4A). If not the full-length protein, but a subsequence, is used for antibody production [43], the epitope might not actually be located on the surface but buried inside the core (Fig. 4B). Moreover, for a sandwich assay approach the position of the epitopes of capture and detection antibody to each other needs to be verified to ensure no spatial hindrance (Fig. 4C). Identical, overlapping or adjacent epitopes would lead to competitive binding between capture and detection antibody and hence the signal detected would be negatively affected. Those same considerations are also necessary for adequate immunogenic peptide selection to ensure its accessibility in the native protein.

Molecular weight is a basic indication of protein size. A simple tool for its computation is provided on the ExPASy Server [58]. It uses UniProt ID or protein sequence as input and can also be used for domain-, region-, or fragment-specific calculation of molecular weight. If a protein is very small, it might simply not have a sufficiently large ASA to bind two antibodies simultaneously. Thus, it has been suggested in literature that a molecular weight of at least 6 kilodaltons (kDa) is required to use a protein as the antigen in a sandwich ELISA [30]. However, assays have been established for smaller molecules, e.g., the AD-implicated amyloid-$\beta$ 42 has a weight of only 4.52 kDa [103]. Molecular weight is thus a limited measurement of a protein’s suitability as an assay target as it is not providing complete information on the available surface for binding.

Ideally, the researcher has access to the experimentally solved protein structure to investigate its overall arrangement: a globular protein will contain many buried residues inside its core, while a more extended protein shape allows a larger portion of the residues to be potentially involved in antibody binding. The solved protein structure can also be used to map the epitope or a potential immunogen onto it to infer its location within the 3D structure and thus its accessibility for the binding antibody.

Experimentally solved structures of proteins and protein complexes are collected and curated in the Protein Data Bank (PDB) [59, 60]. Entries from the database are assigned a unique ID and can be downloaded in a standardized format as a PDB file. Structure-based methods usually require either a PDB ID or file as input. While novel solved structures are continuously deposited to the PDB, currently not even 18% of the residues of the human proteome are covered by experimental structure determination [104]. A protein will therefore very often have only a partially solved structure or might be entirely unresolved. Nevertheless, protein structures can also be generated using homology modeling. This approach uses a template structure with a similar sequence to infer the structure of the protein of interest [105]. One widely used method that offers this service as a webserver is SWISS-MODEL [61] which also provides a linked database of predicted structure models [62]. SWISS-MODEL homology models have expanded the residue coverage of the human proteome towards 50% [104].

Recently, a major advance has been made within protein structure determination due to the release of AlphaFold, a protein structure predictor that frequently achieves accuracies at the level of experimental methods [63]. Alongside the predictor, a database of structure predictions, the AlphaFold Protein Structure Database, has been published which offers almost full coverage of the human proteome [64]. These prediction models can be downloaded in PDB format and used as input for structure-based prediction tools. Similarly to homology models, the accuracy of the predicted protein structures has to be evaluated, which is specified by the model’s confidence. Further, the accuracy of AlphaFold’s surface accessibility prediction has not been benchmarked yet. An in-depth perspective for biologists on the application of the database as well as its limitations is available [106].

While the mentioned protein structure databases (PDB, SWISS-MODEL repository and AlphaFold Protein Structure Database) include incorporated 3D structure visualization tools, dedicated web-based structure viewers exist offering further functionalities. For instance, Mol* (“Mol star”) is the standard viewer incorporated into the PDB and is also available as a stand-alone tool [65]. It takes either a PDB ID or PDB file as input.

To evaluate local (secondary) protein structure and surface accessibility, informative structural properties can also be simply predicted from the protein sequence. These predictions are often less accurate but are available for any protein or peptide for which the sequence is known. Commonly predicted characteristics include ASA, secondary structure and disorder, which can offer valuable insight into the actual surface area that would be available for antibody binding in the immunoassay. A comprehensive collection of structural protein features can be most efficiently acquired from services such as PredictProtein or NetSurfP-2.0. PredictProtein is a broad prediction service with over 30 tools of different structural and functional protein features incorporated [66]. NetSurfP-2.0 predicts ASA, secondary structure and structural disorder from protein sequence and has reported accuracies of 80% and 85% for ASA and secondary structure prediction, respectively [67]. DescribePROT is a database containing over 1.3 million protein entries for which 13 different properties were predicted [68]. All three resources offer easy-to-use web servers, output predictions per residue, and display results as informative plots.

Intrinsically disordered regions, i.e., regions in the protein lacking a defined folding, are an important property to consider. These regions can take various configurations in solution, and do not fold into a unique structure that can be determined experimentally. Such regions have also been shown to highly overlap with the low confidence prediction regions of AlphaFold [107]. As most residues in disordered regions will not fold into a compact structure, most residues will be exposed to the solvent, and can provide a large surface for specific binding, i.e., they make suitable epitopes. Indeed, a study by MacRaild et al. showed epitopes in disordered regions to be smaller and more efficient in their antibody binding compared to structured region epitopes [108]. Information specifically on protein disorder has been collected and made available in several databases. DisProt contains curated annotations of disordered protein regions [69]. All entries have been confirmed experimentally and were collected from scientific literature. MobiDB collects both curated and derived annotations and predictions from various sources and provides a disorder consensus for a protein of interest [70]. Many sequence-based disorder predictors have been developed and their performance has been reviewed elsewhere [109]. Several methods achieve an area under the curve (AUC) above 0.9, demonstrating the reliability of some of these sequence-based disorder predictors [109]. One recent example is IUPred3 [71].

### Proteoform complexity

While it is now established that the human proteome is made up of approximately 20.000 proteins, this does not capture the full extend of the proteome diversity as splicing, protein cleavage and post-translational modifications (PTMs) create various protein variants that stem from the same gene [110].

Researchers need to be aware of the complexity in which the biomarker candidates exist in the human body and establish which proteoforms are of interest for the specific research question.

It should be established if the aim is to develop an immunoassay capable of detecting all variants of the protein target or a specific subset, e.g., one splice variant. Additionally, the knowledge available on existing proteoforms is important to facilitate optimal antibody and immunogenic peptide choice as potential obstacles, e.g., a PTM located in the epitope region, can strongly affect the binding of an antibody.

About 95% of mammalian genes are affected by alternative splicing after the transcription to mRNA, resulting in multiple protein products derived from the same gene [111]. The use of specific isoforms or their ratios as protein biomarkers is gathering increasing attention [111, 112]. This is especially relevant for the biomarker tau protein associated with several diseases termed tauopathies. Six different isoforms of tau exist in the human brain and the relative abundance of these isoforms has been shown to be altered in disease indicating that tau isoform ratios are suitable biomarker candidates [113]. Isoform-specific antibodies and immunoassays have been developed for tau [114] as well as other proteins [115, 116]. Other work has focused on developing assays that explicitly detect all known isoforms of a protein target [117]. Isoform specificity is often not reported for commercial antibodies and is difficult to determine [118]. While additional bands in western blotting might confirm the existence of splicing isoforms, the absence of bands may be explained either by the absence of the isoform from the sample or by the inability of the antibody to recognize the isoform. Note that the exact location of the epitope with respect to the canonical reference sequence may strongly affect the ability of an antibody to recognize a specific isoform.

UniProt provides besides the canonical reference sequence of each protein also the isoforms arising from alternative splicing.

Similarly, many proteins undergo proteolytic cleavage. Often only the cleaved fragment might serve as a biomarker; examples include the AD biomarkers amyloid-$\beta$ and neurogranin [103, 119]. If a fragment is to be detected by an assay, the location of associated cleavage sites is thus essential. Information about a protein’s proteolytic processes, any known cleavage sites and the resultant cleavage products of proteins can be found in UniProt.

PTMs are receiving increased attention because of their possible involvement in various diseases [120–122]. Antibodies capable of recognizing modification-specific proteoforms are therefore of high interest within biomarker assay research. A well-established example is the tau protein on which numerous PTM sites, most importantly phosphorylation sites, haven been identified. Tau’s hyperphosphorylation is regarded as a hallmark process in AD pathogenesis [26]. The use of ELISA to quantify the concentration of total tau and its phosphorylated forms has been established firmly [5] and shows that antibody-based assays can be used for the differentiation of modified and unmodified protein forms. The specificity of PTM-specific antibodies however has been questioned [123, 124] and validation is highly necessary. If unaware of an existing PTM within or close to a biomarker’s epitope, antibody-binding could be negatively affected (Fig. 3B) [125–127]. Therefore, awareness of the potential modification of residues is needed when examining epitopes or choosing immunogenic peptides to either allow precise recognition of the modified protein form or limit the chance that PTMs negatively affect the assay. Known PTMs of a protein can be examined in depth through database searches. UniProt contains many annotations for modified residues but PTM-specific resources are available as well. PhophoSitePlus is a curated database of experimentally confirmed modification sites [72]. It provides a graphical overview of the type, position, and amount of evidence for each modification. iPTMnet collects isoform-specific annotations from multiple sources and assigns scores to each PTM based on the available evidence [73]. Various PTM prediction methods have also been developed; most are focused on one specific type of modification. In contrast, MusiteDeep is an online tool that allows prediction of many PTM types given a protein sequence as input [74]. The predictor achieves AUC values between 0.732 and 0.993 depending on the PTM type. Further PTM resources and modification-specific predictors have been reviewed elsewhere [128].

### Protein interactions

Proteomics studies have identified over 3000 proteins in CSF [129] and over 6000 proteins in the plasma proteome [130]. Hence, it is very likely that proteins interact with other molecules present in the sampled body fluid (e.g., protein, nucleotides or metabolites). Potential interactions need to be considered to eliminate the possibility that binding molecules will hinder the biomarker detection in immunoassays. An investigation of the surface regions affected by intermolecular interactions and aggregation propensities can help exclude antibodies with an unfavorable epitope or decide on an immunogenic peptide unaffected by those interactions to ensure successful antibody-binding.

The formation of protein complexes is vital for most biological functions. If the interface of a protein interaction site is identical or overlapping with an epitope or immunogenic peptide, the antibody binding might be hindered (Fig. 3C). Many studies have been performed to analyze and predict the binding of proteins to other proteins, nucleic acids or ligands [131]. The previously mentioned tools PredictProtein [66] and DescribePROT [68] also incorporate predictors of interacting residues. Additionally, DescribePROT includes the molecular recognition feature (MoRF) predictor MoRFchibi [132]. MoRFs are protein-binding regions within intrinsically disordered regions which are often capable of binding more than one partner [133, 134]. Several other sequence-based predictors have been developed for this particular task and a wide selection has been reviewed by Katuwawala et al. [134]. Stand-alone tools also exist in this field: HybridPBRpred is a recent web-based predictor of protein-binding residues [75]; ANCHOR2 is available for the prediction of protein binding regions specifically in disordered proteins [76].

Databases also provide useful information. InterPro [77] is a well curated domain database that supports identification of known binding domains. The disorder database MobiDB [70] is also an excellent resource to identify and examine binding sites in disordered regions: it links knowledge about interactions from PDB complexes to the protein sequence, it provides information on the conformational transitions occurring within disordered binding sites, and it also includes curated annotations from the disordered binding site (DIBS) and eukaryotic linear motifs (ELM) databases [135, 136].

Protein aggregation and oligomerization have high importance within the pathogenesis of several neurodegenerative diseases [137]. The accumulation of proteins as well as the prior formation of oligomeric species is one of the hallmarks of various neurodegenerative diseases [138–140] and a strong research effort exists to analyze these protein aggregates by antibody-based detection methods [141]. Many conformation-specific antibodies and immunoassays have been developed [141–143]. Lu et al. used a process of solubilizing neurofilament aggregates before measurement by ELISA [144]. Independent of the desired approach to handle accumulated proteins, researchers need to be aware that oligomerization and aggregation of a protein can easily cover epitopes (Fig. 3D). This could lead to the protein concentration being grossly underestimated [145]. Solubility and aggregation are closely and inversely related; both properties can be predicted by Aggrescan3D 2.0 given a 3D protein structure [78]. To predict solely aggregation propensity, PASTA 2.0 is a sequence-based alternative that can highlight the aggregation prone regions within an amino acid sequence [79]. It might also be advisable to browse if a protein is included in the amyloid database AmyPro [80] which collects confirmed amyloidogenic protein fragments and regions.

### Epitopes

Epitope localization and characterization may support a researcher’s epitope-specific antibody survey and selection if information from the manufacturer is insufficient. Likely epitope residues can be predicted and thus a more focused examination of the probable binding region (and its potential issues regarding antibody access) can be performed. As often at least a broad immunogenic region is provided for commercial antibodies, it might be helpful to compare that knowledge with the results derived from the bioinformatic prediction tools. Additionally, epitope related tools can be a convenient way to support immunogenic peptide selection as residue stretches predicted as epitopes will most likely constitute a suitable peptide for immunization as well.

**Fig. 5:**
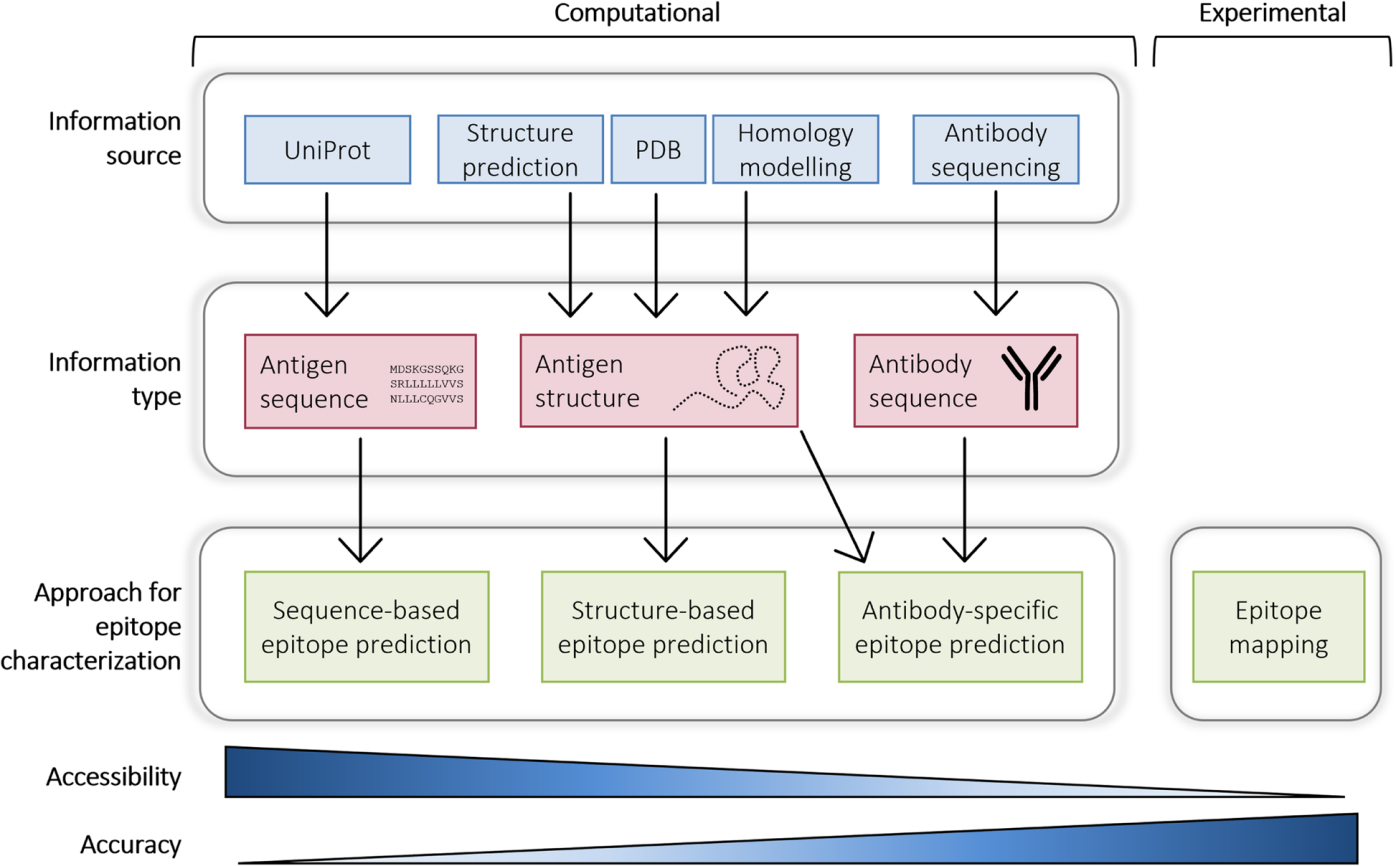
Available computational and experimental approaches for epitope characterization. Next to epitope mapping, several bioinformatics tools exist for epitope prediction. Bioinformatics information sources (blue) give access to different types of information (red). Sequence-based, structure-based and antibody-specific epitope predictors exist that differ regarding the required input data; methods that require more effort usually offer more accurate results

Figure 5 illustrates the different approaches available to characterize the epitope or epitopes of an antigen. While, compared to epitope mapping, computational approaches offer a more time- and resource-saving strategy to learn about an antigen’s potential epitope regions, they are less reliable. Various tools exist that differ in the required input and the accuracy of their prediction.

The most widely applicable tools are sequence-based epitope predictors as only the protein sequence is required and many methods have been published with slightly differing outputs. BepiPred-2.0 is a widely cited sequence-based predictor for linear epitopes of different length [81]. The tool calculates a probability score for each residue and displays the residue stretches meeting an adjustable threshold. For prediction of conformational epitopes from sequence SeRenDIP-CE reported an AUC of above 0.7 [82]. Structure-based epitope predictors usually outperform sequence-based approaches in accuracy but are limited in their capacity as a protein structure needs to exist [146]. Two examples are ElliPro [83] and epitope3D [84].

The aim of the applications mentioned hitherto is the identification of surface residues that have the potential to be recognized by antibodies. It might be difficult to determine which amino acids would ultimately comprise the epitope for the antibody of interest if many residues are predicted as epitopes [147]. A proposed solution for this concern is antibody-specific epitope prediction. Such methods attempt to predict one distinct epitope on an antigen given additional information about the antibody to bind [147]. One such tool is EpiPred, part of the antibody prediction toolbox SAbPred [85, 86]. EpiPred requires both antigen and antibody structure but has also shown that its performance is not significantly affected when using antibody homology models instead of solved 3D structures [85]. SAbPred also provides an antibody-specific homology modeler, ABodyBuilder [148]; it requires the antibody sequence as input and the outputted model can then directly be used for EpiPred.

Other epitope resources to be aware of are the Immune Epitope Database (IEDB) [87] and the Structural Antibody Database (SAbDab) [88]. The IEDB collects confirmed epitopes from experimental data and allows to search the database for specific organisms, antigen, host and epitope type. The IEDB also offers the Immunome Browser: epitope data is mapped onto the protein sequence to allow easy identification of regions tested as epitopes. SAbDab collects all solved antibody structures in the PDB and provides coherent and consistent annotations for them.

The uniqueness of an epitope or potential immunogenic peptide is an important characteristic to be assessed. If an antibody can bind a structure other than the intended biomarker candidate, a false positive signal would be the result. Non-specific binding by commercially available antibodies leading to erroneous immunoassays has been reported in many scientific publications [149–151]. Off-target binding is thus still an active source for concern when working with commercial antibodies. Epitope specificity can most easily be predicted if the immunization was carried out with a small peptide, as the resulting epitope will most likely be a linear sequence. Basic Local Alignment Search Tool (BLAST) searches extensive sequence databases for regions with high sequence similarity to an input sequence [89]. This tool is therefore ideally suited to identify regions in the proteome that are highly identical to the epitope region of the antigen and might result in off-target antibody binding. If BLAST indicates several highly aligned protein sequences to the linear epitope, the corresponding antibody might not be suited to dependably bind only the antigen of interest. If the full-length protein was used for immunization, the epitope may be conformational. There is currently no good method to predict the specificity of conformational epitopes.

### Immunoreagent databases

The challenge to search for the most advantageous commercial immunoassay kits or antibodies is a daunting task. A widely used approach of purchasing many antibodies and kits and testing them in parallel is expensive and time consuming with no guarantee for success [39]. Frequently, one particular antibody is offered under multiple catalog numbers by different vendors. Antibodies are often not validated for the desired application or sample type, or the validation has not been performed rigorously enough [38, 46]. This leads to many commercial antibodies not performing adequately, and their unreliability has been recognized as one of the major contributor to the reproducibility crisis of research [39, 46, 152]. For instance, as part of the HPA project 20.000 commercially available antibodies have been tested for their use in immunohistochemistry, with less than half of them performing acceptably [153].

The most convenient and thorough way to survey available and find trustworthy immunoreagents is the use of antibody validation databases. These collections contain more objective and reliable antibody and immunoassay evaluation data. The product range from multiple suppliers is gathered to be easily compared and the criteria for validation data are often more stringent. Several of those databases are described below and summarized in Table 3.

Antibodypedia [154] provides a catalog of antibodies against human proteins and their available validation data. Submission of antibodies and their associated validation is open to everyone but must meet the portal’s criteria and is reviewed before publishing. Furthermore, validation data is always application specific and the search for antibodies can be filtered according to the desired experiment setup. Antibodypedia assigns application-specific scores to each antibody based on the available data, thereby providing a trustworthy and thorough assessment for researchers. A different approach of antibody evaluation is offered by CiteAb [155]. This database ranks antibodies according to the number of peer-reviewed publications that have cited it. This allows the identification of the antibodies most trusted in the research community as well as a cross-reference to published validation information. The Antibody Registry is part of an effort within research reproducibility, the Resource Identification Initiative [156]. The aim of this initiative is to provide all used material with a Research Resource Identifier that can be used to improve reporting in scientific publications and thereby increase reproducibility of experiments. The Antibody Registry assigns this permanent identifier (Antibody ID) to every antibody, allowing researchers to find the associated publications for every specific antibody based on its ID. It also provides the proper citation style for each antibody. Further guides include antibodies-online which provides standardized product information and also ranks their range based on available validation data, and Biocompare which allows easy filtering and comparison across suppliers.

With this multitude of information sources available a more exhaustive survey of antibodies and immunoassay kits can be performed. By letting researchers more easily identify trustworthy immunoreagents (and avoid unreliable ones) for their specific application, antibody databases can help manage the unreliability problems of research antibodies. Still, rigorous antibody validation by the researchers themselves is indispensable [38], and several excellent guidelines for the best suited antibody validation process exist [157, 158].

**Table 3:** Immunoreagent databases. Selection of online databases and catalogs of affinity reagents such as antibodies and immunoassay kits. Information on the provided filter options, number of antibodies and companies presented on each website, and website link are specified

Database	Filter options	Antibodies	Companies	Website
Antibodypedia	Antibody type, application, conjugate, host, reactivity, validation method	>4.5 million	98	https://www.antibodypedia.com/
CiteAb	Antibody type, application, clonality, conjugate, host, modification, mutation, reactivity, validation method	>5.7 million	280	https://www.citeab.com/
Antibody Registry	Clonality, clone ID, host	>2.5 million	>500	https://www.antibodyregistry.org/
antibodies-online	Antibody type, application, binding specificity, clonality, conjugate, host, isotype, reactivity	>4 million	>250	https://www.antibodies-online.com/
Biocompare	Antibody type, application, clonality, conjugate, host, isotype modification, reactivity	>3.7 million	142	https://www.biocompare.com/

## Use cases

To illustrate the use and interpretation of the bioinformatics tools and data resources introduced in this review, we present use cases for three proteins that are either established biomarkers or promising candidates for AD: neurogranin, tau and TREM2. These proteins were selected to cover a wide range of possible outcomes of the bioinformatics analysis. As this is a retrospective study of known or potential biomarkers, there is obvious bias; the analysis leading to the conclusion that these proteins are interesting candidates for AD is thus unsurprising. Nevertheless, we still expect that these use cases will serve as helpful examples how to interpret predictions from bioinformatics tools and annotations from data resources. The complete use cases are provided as an appendix to this review (see Additional file 1). Here, we shortly highlight the results we considered most interesting considering the established knowledge about these proteins, specifically as immunoassay targets.

### Neurogranin

Based on predictions of bioinformatics tools and annotation in data resource neurogranin appears to be an optimal biomarker candidate. The analysis of its biological context shows a strong association with the brain and AD. A detailed analysis of the protein’s structure reveals that the protein is generally stretched-out and shows a mostly natively disordered protein structure. Hence, there is a relatively large surface area available for antibody binding despite the low molecular weight of the protein. Many potential obstacles for successful immunoassay development do not seem to be relevant for neurogranin, e.g., prediction shows a low probability to aggregate and there are no known isoforms. During the computational analysis the C-terminus emerges as the most suitable site for antibody binding. The assessment of neurogranin by bioinformatics tools agrees with research findings. Indeed, three successful neurogranin immunoassay have been developed with the majority of the antibody epitopes being located in the C-terminal region [119, 159, 160]. All three assays have been compared by Willemse et al. establishing high correlation of the assays between each other [161]. One identified cause for concern is the high sequence similarity between neurogranin and neuromodulin within the IQ domain both proteins contain. Interestingly, the expected cross-reactivity with neuromodulin of antibodies binding the IQ domain has been confirmed in a recent study [119]. Retrospectively, the information collected through bioinformatics resources and tools could have guided researchers towards the development of antibodies with favorable epitopes.

### Tau

Examination of predictions and annotations for tau exposes various obstacles to the successful immunobased detection. Tau has been found in EVs and it is predicted to contain several aggregation hot spots. In addition, tau has a variety of proteoforms because of its many different splice variants and PTM sites. In terms of structure, the predictions may be more difficult to interpret. The AlphaFold model of tau has a generally stretched out structure, with very little inter-residue contacts; there is only one helical region. This agrees with previous studies aiming to characterize the structure of tau [162], where it is indeed found that tau in its soluble form is not compactly folded, but an ensemble of different configurations with transient secondary structures. In its native form, tau forms a molten-globule like state; it is therefore more difficult to predict which residues are available as a binding surface for an antibody. Nevertheless, tau is well-established and extensively studied as an immunoassay target. Tau as a biomarker affirms that identified points of caution can be taken into account during immunoassay implementation. The existence of phosphorylation sites and alternative splicing isoforms of tau is firmly verified and proven to be tightly associated with the pathogenesis of various tauopathies [26, 113]. This has indeed been successfully exploited to develop tau proteoform specific assays that are used for clinical diagnosis [5]. Measuring tau in EVs as a biomarker is also actively pursued [163]. The computational analysis identified tau as a protein prone to aggregation. Aggregation of tau is indeed known to be one of the hallmarks of AD pathogenesis [164].

### TREM2

TREM2, or more specifically its soluble form (sTREM2) which is comprised of its extracellular region, is comparatively less well established as an AD biomarker. It is much less specifically expressed in the brain, however, annotations show a clear connection to the brain, to CSF, and to AD. Much of the sequence of sTREM2 is part of the Ig-like domain. As this domain might be involved in binding an interaction partner and is not unique to the TREM2 protein, it does not constitute a good epitope for a TREM2-specific antibody. The C-terminal region of sTREM2 seems to be a more suitable region for antibody binding. Several predictions for TREM2 have to be considered carefully. The AlphaFold model of TREM2 highlights the limited accuracy for inter-domain positions considering the relative positions between the transmembrane helix and the extracellular domain. The aggregation prediction by Aggrescan3D 2.0 relies heavily on the hydrophobicity of surface residues. The region of highest aggregation propensity thus corresponds to the highly hydrophobic transmembrane region; however, this region is not present in the soluble form. The bioinformatics analysis of TREM2 exemplifies that it is important to consider if the predicted annotations actually fall within the matrix-specific protein product.

## Conclusions

The cross-technology translation gap between MS and antibody-based assays continues to be a major bottleneck within the biomarker development and thus a major factor limiting the successful clinical implementation of fluid biomarkers. Therefore, optimization and rationalization of biomarker assay design should receive increased attention. In this review, we examined the typical workflow of novel immunoassay development to identify steps that can benefit from the incorporation of bioinformatics tools. We determined areas of interest during the selection of appropriate biomarker candidates, antibodies and immunogenic peptides. For each area of interest we established which specific properties could be investigated with the aid of online databases, prediction and visualization tools. For each property of interest we discussed at least one specific tool and illustrated how the gained knowledge can enhance or accelerate the assay development process.

Recent progress within bioinformatics has led to the release of a vast number of resources useful for fluid protein biomarker research. The value of these tools is constantly increasing as new entries are added to databases, the performance of prediction tools is improved, and the integration across different databases is promoted. Especially the release of AlphaFold and its corresponding database is expected to strongly increase the prediction accuracy of methods dependent on protein structures, e.g., epitope predictors. Note that while the AlphaFold Structure Database provides researchers with a protein structure model for every human protein, the use of these structure models in structure-based predictors should be gauged for proteins with a high disorder content. As disordered regions are defined by the absence of a definite structure, prediction on structures of highly disordered proteins are not meaningful and the use of sequence-based prediction tools is advisable for such regions.

Much effort has been put into making these tools user-friendly for the general researcher. However, incomplete knowledge about which types of resources exist and which would be most suitable for the matter at hand might discourage many researchers from implementing computational methods in a meaningful way. Hence, with this review, we aim to present the scope of current available bioinformatics tools and provide explicit ideas on how to utilize them for biomarker assay development. Especially the included use cases on AD biomarker candidates offer an easy demonstration of how to use the presented tools and resources and critically evaluate the findings.

While bioinformatics have the potential to save time, resources and money, the limitations of these computational resources should be contemplated, especially when basing decisions about the prioritization or exclusion of biomarker candidates and antibodies on them. The accuracy of the results can vary greatly between prediction tasks. For instance, while an independent benchmark study of sequence-based disorder predictors reported AUC scores up to 0.957 [109], current sequence-based epitope predictors report lower performance measures between 0.62 and 0.704 [81, 82]. The difficulty of the prediction task should therefore always be considered when examining results. Additionally, databases can contain bias towards well-studied proteins. Proteins that have been investigated thoroughly have extensive annotations, while the so far less significant part of the proteome might be missing many observations. It is important to remember that the absence of annotations might not present the actual state of a protein, e.g., missing PTM annotations in a database give no guarantee of this protein not being modified.

In conclusion, we expect this review to provide a valuable introduction into bioinformatics solutions for the current challenges within the biomarker assay development pipeline. The collection of suitable tools compiled and categorized here provides a starting point to incorporate the methods and can save time and resources.

## Supplementary Information

**Additional file 1:** Use cases for dementia protein biomarkers. PDF document of extended use cases for three biomarker candidates of Alzheimer's Disease: neurogranin, tau and TREM2. For each biomarker a suitability survey was performed and then compared to the current knowledge of these proteins. The document contains figures and results of the used bioinformatics tools and data resources as well as our interpretation of these results.

## Data Availability

Not applicable.

## Abbreviations

AD: Alzheimer’s Disease
ASA: Accessible surface area
AUC: Area under the curve
BLAST: Basic Local Alignment Search Tool
CSF: Cerebrospinal fluid
ELISA: Enzyme-linked immunosorbent assay
EV: Extracellular vesicle
GO: Gene ontology
HPA: Human Protein Atlas
IEDB: Immune Epitope Database
kDa: kilodalton
MoRF: Molecular recognition feature
MS: Mass spectrometry
OMIM: Online Mendelian Inheritance in Man
PDB: Protein Data Base
PTM: Post-translational modification
SAbDab: Structural Antibody Database
