# Supplementary material for: Bioinformatics tools and data resources for assay development of fluid protein biomarkers
Source: Biomark Res. 2022 Nov 15;10:83. doi: 10.1186/s40364-022-00425-w (PMC9667682; doi:10.1186/s40364-022-00425-w)
Supplement: Supplementary file 1 — Additional file 1: Use cases for dementia protein biomarkers. PDF document of extended use cases for three biomarker candidates of Alzheimer's Disease: neurogranin, tau and TREM2. For each biomarker a suitability survey was performed and then compared to the current knowledge of these proteins. The document contains figures and results of the used bioinformatics tools and data resources as well as our interpretation of these results. [file 40364_2022_425_MOESM1_ESM.pdf]

Use cases for dementia protein biomarkers

Supplement to: Bioinformatics tools and data resources for assay development of fluid protein biomarkers

Katharina Waury, Eline A.J. Willemse, Eugeen Vanmechelen, Henrik Zetterberg, Charlotte E. Teunissen and Sanne Abeln

To illustrate the use and interpretation of the bioinformatics tools and data resources introduced in the review, we present use cases for three proteins that are either established biomarkers or promising candidates for the most common cause of dementia, Alzheimer's Disease (AD): neurogranin, tau and TREM2. These proteins were selected to cover a wide range of possible outcomes of the bioinformatics analysis. We aimed to show the different circumstances in which supportive information is attained, and in which obstacles or points of caution are identified. There are also cases in which the bioinformatics tools offer no, inconclusive or contradictory results as not every tool is expected to be useful for every context. As this is a retrospective study of known or potential biomarkers, there is obvious bias; the analysis leading to the conclusion that these proteins are interesting candidates for AD is thus unsurprising. We still expect these use cases to be helpful and supportive for biomarker researchers interested in incorporating bioinformatics tools and data resources.
The entry for each protein begins with background into its current status as an AD biomarker. This is followed by a selection of the bioinformatics analysis results which were deemed to be most interesting. Where possible, we reflected on the agreement of the analysis results with published research and possible shortcomings of these analyses. The summary is followed by the in-depth analysis of the protein, ordered by the area of interest and the method or database used. Corresponding references and links where possible are provided. The conclusions for every area of interest are summarized in an "interpretation" box which summarizes the outcome of each analysis step by colour as:

- Supportive information
- Point of caution
- Potential obstacle
- Inconclusive/No data available

Neurogranin

Summary

Background

Neurogranin is a 78 amino acid-long protein expressed by neurons that is involved in learning and memory processes [1]. Neurogranin has been proposed as a novel biomarker of synaptic dysfunction or damage for several neurological diseases [2]. The protein has been most thoroughly investigated as a potential cerebrospinal fluid (CSF) biomarker for AD and several studies have shown an increased neurogranin concentration in CSF compared to controls and other neurodegenerative diseases [3, 4].

Bioinformatics analysis

Many biological aspects were observed for neurogranin that strengthen its potential as a fluid biomarker for AD. Supportive data exists in several areas of interest: protein function, interaction partners and tissue-specific expression. These findings would have

likely stood out during a suitability study of protein candidates and lead to the inclusion of neurogranin as a promising biomarker.

While the small size of neurogranin and its known C-terminal cleavage fragment are a point of caution, a more detailed analysis of the protein's structure revealed the relatively large surface area available for antibody binding despite the low molecular weight. The generally outstretched fold and the high disorder content of the protein sequence leave barely any residues buried and thereby inaccessible. The confidence in the available protein structure prediction by AlphaFold is strong, as it agrees highly with other information collected from a partly solved protein structure of the PDB, sequence-based structural feature prediction and disorder annotations and predictions.

Many potential obstacles for successful immunoassay development do not seem to be relevant for neurogranin, e.g., there is a low probability to aggregate and no known isoforms. During the analysis the C-terminus appeared to be the most suitable site for antibody binding as this region is not a domain or predicted interaction site and is highly outstretched because of the disordered conformation. Accordingly, this region was also identified by BepiPred-2.0 and ElliPro as a likely epitope region.

Reflection

Several successful neurogranin immunoassay studies corroborate this bioinformatics analysis. Nazir *et al.* developed a sandwich assay using two antibodies with well-defined epitopes [1]. Both antibodies used in this assay are binding to the C-terminus region of the protein, specifically at amino acids 52-63 and 63–77, i.e., the region we identified as most appropriate to allow unhindered antibody binding. The neurogranin Erenna® assay of Washington University utilized a capture antibody binding the C-terminus (amino acids 49-60) and a detection antibody binding the also disordered N-terminus (amino acids 11-23) [5]. A third established assay by ADx combined a C-terminal antibody (amino acids 53-64) with an antibody specifically recognizing a truncated form of neurogranin at the C terminus as well [6]. All three assays have been compared by Willemse *et al.* establishing high correlation of the assays between each other [7]. Interestingly, the expected cross-reactivity with neuromodulin of epitopes located in the IQ domain was already confirmed in a recent study [1]. Retrospectively, the information collected through bioinformatics resources and tools could have guided researchers towards the development of antibodies with favourable epitopes.

Protein function

UniProt (Database of annotations) [8, 9]

GO - Molecular function (selection):

- calmodulin binding

GO - Biological process (selection):

- associative learning
- nervous system development
- positive regulation of long-term synaptic potentiation
- postsynaptic modulation of chemical synaptic transmission
- signal transduction

Interpretation

Neurogranin has an established function in the brain, more specifically for learning and memory processes. A malfunction of neurogranin leading to impaired cognitive function, as is the case in dementia, is coherent.

Interaction partners

STRING (Database of annotations, database of predictions) [10, 11]

- Calmodulin-1 (CALM1)
- Calmodulin 3 (CALM3)
- Opalin (OPALIN)
- Zinc finger protein 804A (ZNF804A)
- IQ motif containing J (IQCJ)
- Brain-specific homeobox protein homolog (BSX)
- Neuromodulin (GAP43)
- Neurofilament light polypeptide (NEFL)
- Neugrin (NGRN)
- Visinin-like protein 1 (VSNL1)

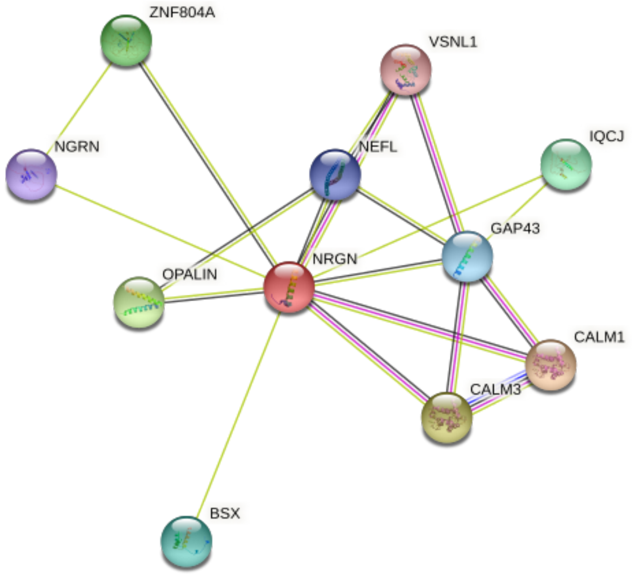

Interpretation
Neurogranin has known interactions with potential biomarkers for AD (e.g., GAP43) and neuronal injury (e.g., NEFL, VSNL1). Its interaction network strengthens the role of neurogranin in AD pathology.

Disease involvement

DisGeNET (Database of annotations) [12, 13]

Neurogranin shows the strongest gene-disease association score for non-dementia diseases, such as schizophrenia and bipolar disorder. However, several publications support the association of neurogranin with AD (31 publications), dementia (5 publications) and neurodegenerative disorders (5 publications).

Interpretation
Literature already exists considering neurogranin as a biomarker candidate for AD and related pathologies.
Potential associations with other brain disorders should be carefully checked to establish the use of neurogranin as an AD-specific biomarker for differential diagnosis.

Tissue-specific expression

HPA (Database of annotations) [14, 15]

Neurogranin shows the highest expression in brain tissue but has also been found in others.

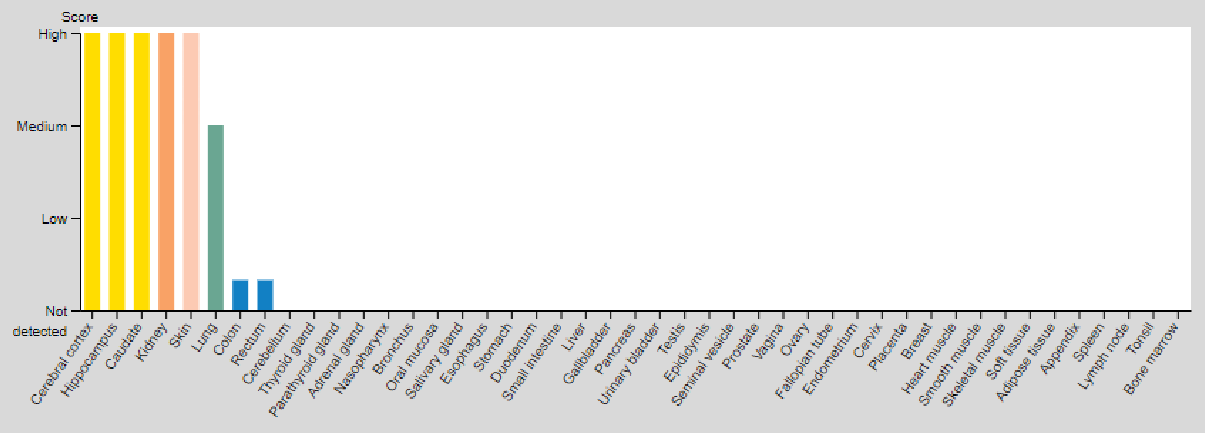

Image credit: Human Protein Atlas

HBFP (Database of annotations) [16]

Body fluids in which neurogranin was identified:

- cerebrospinal fluid (13 papers in total) | confidence score: 0.945
- plasma/serum (41 papers in total) | confidence score: 0.815

Interpretation
Protein expression is quite specific to the brain; thus protein concentration in body fluids will only faintly be affected by expression of other organs than the brain. The presence of neurogranin both in CSF and blood is established.

Subcellular localization

UniProt (Database of annotations) [8, 9]

GO - Cellular component (selection):

- cytosol
- mitochondrial membrane
- nucleus
- postsynaptic membrane
- axon
- dendritic spine head
- glutamatergic synapse

Interpretation
The high expression and localization of neurogranin in brain cells (neurons) is established.

EV localization

Vesiclepedia (Database of annotations) [17, 18]

Neurogranin mRNA was identified in one study of colorectal cancer cell-derived microvesicles but no evidence exists so far on the protein level.

Interpretation
There is no definite evidence for or against EV localization of neurogranin.

Protein structure

Compute pI/Mw (Calculation) [19]

Full-length protein (1-78 aa): 7.618 kDa
Fragment (55-78 aa): 1.818 kDa

PDB (Database of annotations) [20, 21, 22]

No fully solved structure of neurogranin is available in the PDB. However, one partly solved structure (PDB ID: 4E50) shows the neurogranin IQ domain peptide (shown in pink) in complex with calmodulin (green). The domain adopts an alpha helix structure.

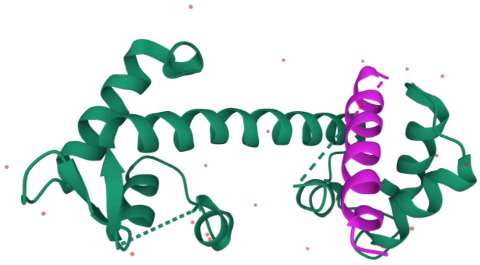

AlphaFold Protein Structure Database (Database of predictions) [23, 24]

The AlphaFold prediction is highly confident about the central alpha helix (in the region of the IQ domain). The other regions towards the N- and C-terminus are almost entirely predicted as disordered regions with lower confidence. Low prediction confidence is expected within disordered regions as no definitive conformation will exist.

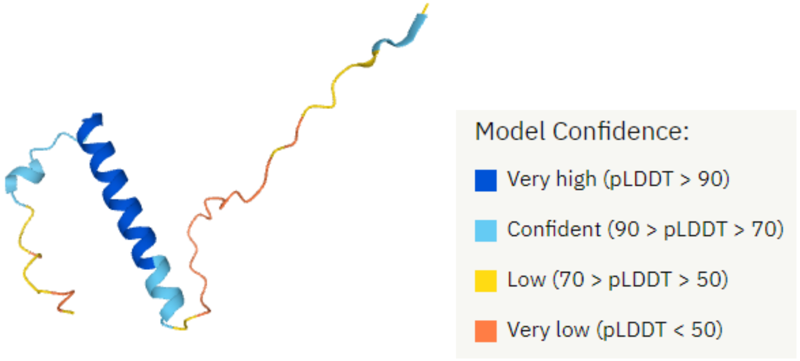

DescribePROT (Database of predictions) [25, 26, 27]

A central alpha helix structure is predicted, the majority of other residues are predicted as coil, i.e., mostly disordered. Only few residues are predicted as buried.

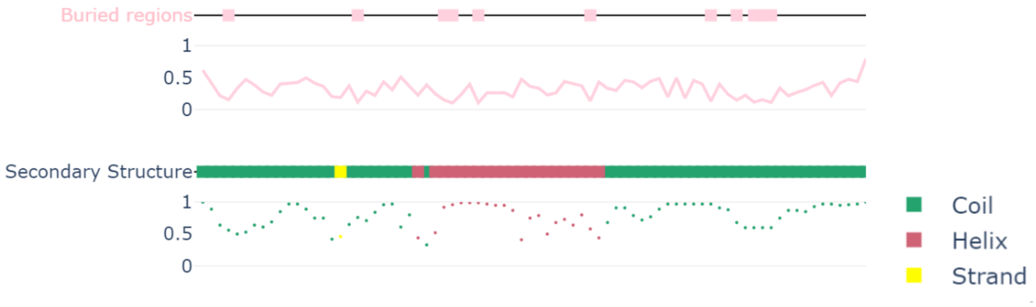

NetSurfP-2.0 (Prediction) [28]
Again, an alpha helix is predicted to lie between amino acids 25 to 45. All other residues are predicted to be disordered and surface accessible.

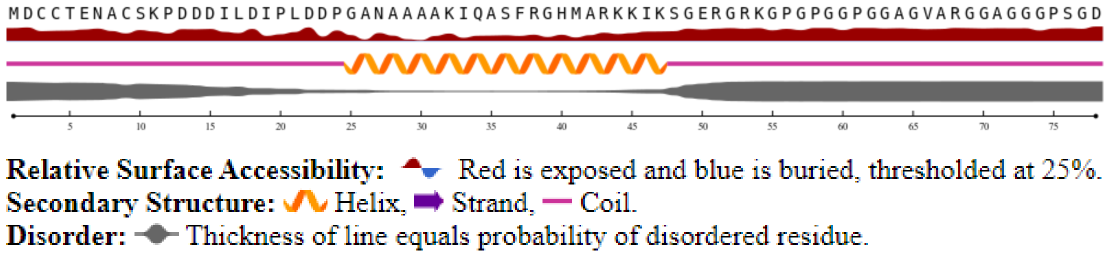

DisProt (Database of annotations) [29]
No entry found.

MobiDB (Database of annotations, database of predictions) [30, 31]
The highest disorder prediction can be found at the C-terminus.

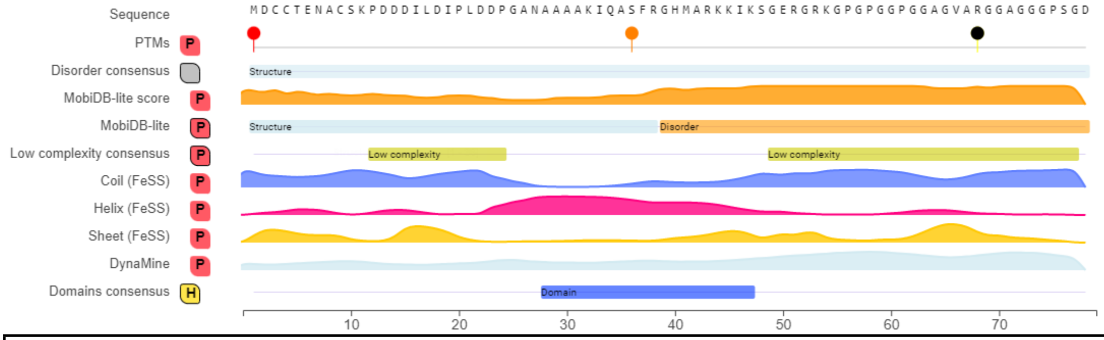

Interpretation
Neurogranin is a relatively small protein. Especially the specific detection of any fragments might be challenging because of the limited surface area available for binding.
All tools used for the structural analysis of neurogranin show high agreement with each other. The helix conformation of the IQ binding domain found by experimental structure determination is predicted by all bioinformatics tools.
Large parts of neurogranin are predicted to be disordered and surface accessible which provides a greater area for antibody binding despite the small molecular weight.

Isoforms and cleavage products

UniProt (Database of annotations) [8, 9]
One isoform exists which is known to be cleaved into a fragment of amino acids 55-78.

Interpretation
Isoform specificity does not need to be considered during antibody selection.

PTMs

PhosphoSitePlus (Database of annotations) [32, 33]

A few modifications are known across the protein sequence. Especially a methylation site (grey) at the C-terminus has the most evidence associated with it.

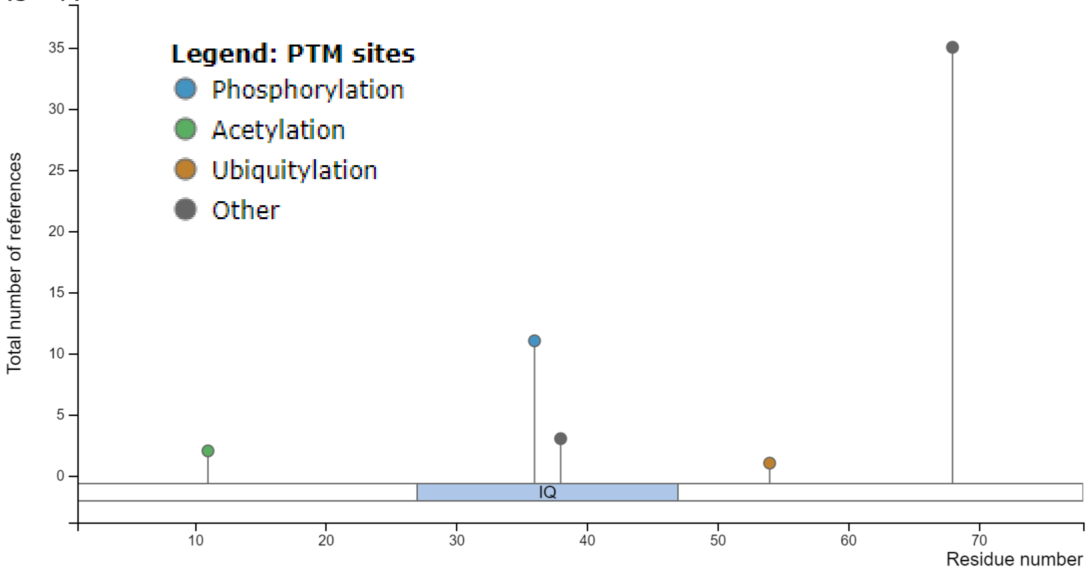

iPTMnet (Database of annotations) [34, 35]

Some modifications are annotated with low to medium confidence scores.

Site<br>All	PTM Type<br>All	PTM Enzyme	Score<br>All
M1	Acetylation		★☆☆☆
S36	Phosphorylation	P05771 (PRKCB) , P17252 (PRKCA) , P05129 (PRKCG)	★★☆☆
R38	Methylation		★☆☆☆
R68	Methylation		★★☆☆

MusiteDeep (Prediction) [36]

The predicted PTM sites overlap with annotations (see S36 and R68), while additional PTMs of less frequent type, e.g. palmitoylation and hydroxylation, are predicted especially towards the termini of neurogranin.

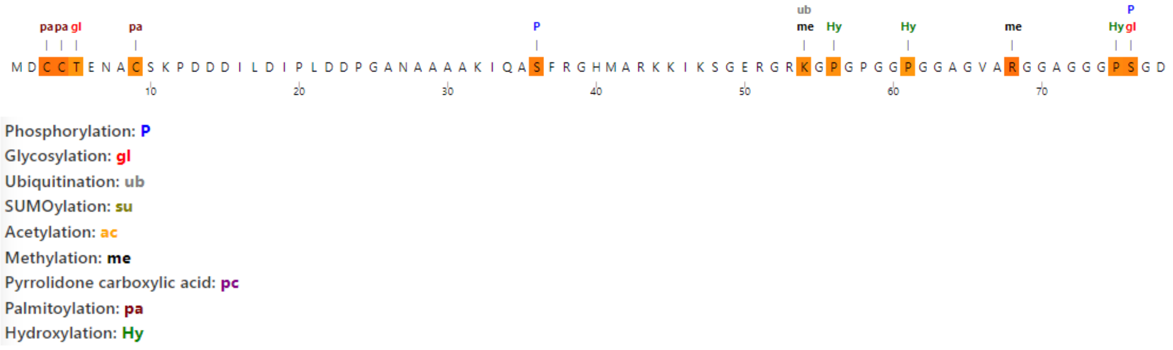

Interpretation

PTM annotations and predictions overlap to give high confidence in some of the modification sites.

Some PTM sites could hinder antibody binding but the coverage of the modifications is unknown.

Interaction residues

DescribePROT (Database of predictions) [25, 37, 38, 39]

No wide DNA- or RNA-binding is predicted for neurogranin; protein binding is predicted towards the N-terminus and the central region. Molecular recognition features (MoRFs) are also predicted in the central region.

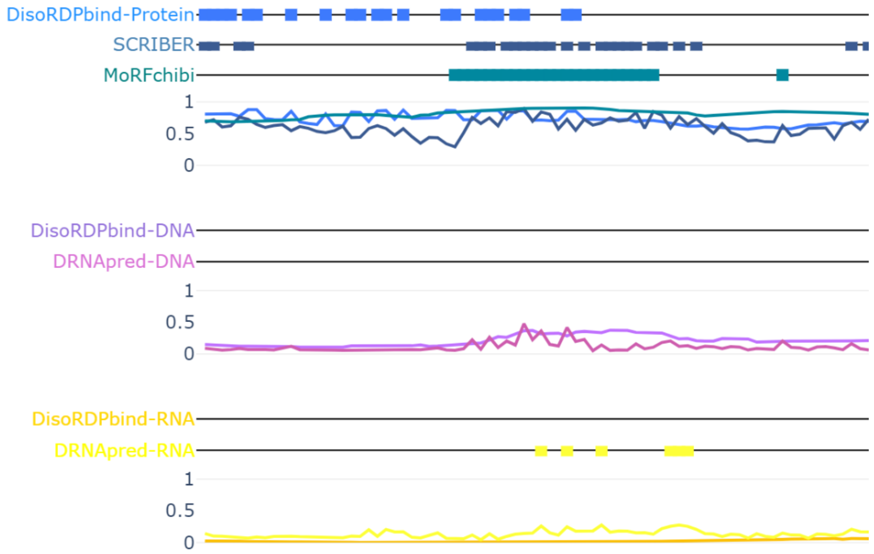

ANCHOR2 (Prediction) [40]

The highest probability for disordered binding regions is predicted in the central region. The lowest probability is found at the N-terminus and between amino acids 50-60.

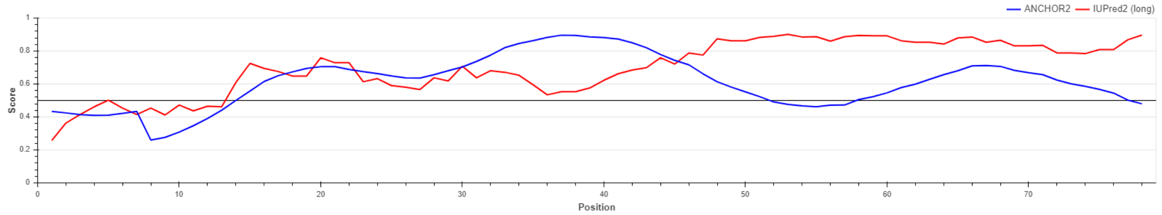

InterPRO (Database of annotations) [41, 42]

The binding site of calmodulin (IQ domain) is annotated within the central region of neurogranin (amino acids 26-55).

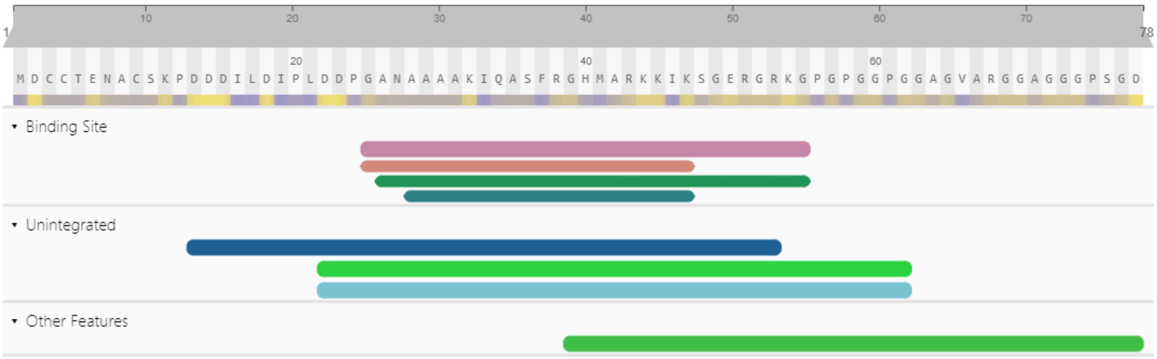

MobiDB (Database of annotations, database of predictions) [30, 31]

No annotation of linear interacting peptides.

Interpretation
Prediction tools give the highest propensity for binding regions in the center of neurogranin which overlaps highly with the annotated IQ domain. This region might not be available for binding an antibody as the domain is known to interact with calmodulin (which is present in body fluids).
The predictors agree on the region between the IQ domain and the C-terminus to be not involved in binding. This might constitute a suitable binding region for antibodies.

Aggregation

Aggrescan3D 2.0 (Prediction) [43]

Some small areas within neurogranin are predicted as aggregation prone residues but generally the protein exhibits a low aggregation propensity.

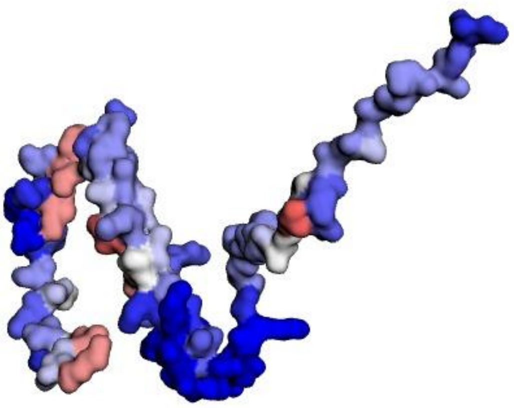

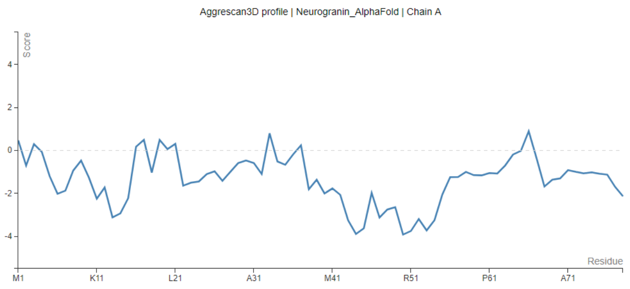

PASTA 2.0 (Prediction) [44]

The sequence-based prediction is generally low and without any aggregation hotspots.

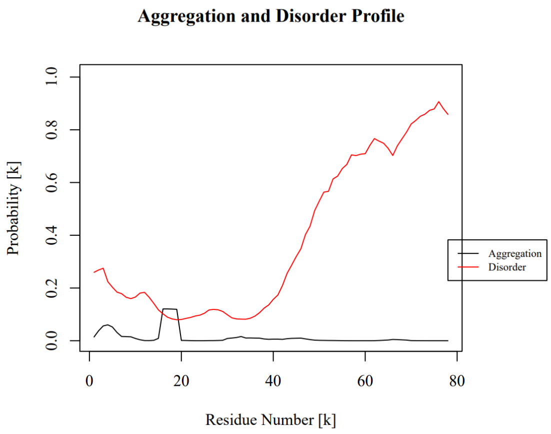

AmyPro (Database of annotations) [45, 46]

No entry. One protein sequence match was found to functional amyloid Ice-structuring protein A, but this seems insignificant.

Interpretation
Aggregation of neurogranin is predicted to be unlikely and would not be an obstacle for antibody-based detection.

Epitope prediction

BepiPred-2.0 (Prediction) [47]

Using the default threshold of 0.5 many residues are predicted as potential epitopes.
Threshold = 0.5

Sequence Markup

```
Epitopes   : .....EEEEEEEEEEEEEEEEEEEEEEE.........EEEEEEEEEEEEEEEEEEEEEEEEEEEEEEEEEEEEEE...
Predictions: MDCCTENACSKPDDDILDIPLDDPGANAAAAKIQASFRGHMARKKIKSGERGRKGPGPGGPGGAGVARGGAGGGPSGD
             1--------10--------20--------30--------40--------50--------60--------70-------
```

With an increasing threshold (0.5 → 0.6) only the disordered C-terminus region (amino acids 45-70) is predicted as an epitope. No residue has an epitope probability score higher than 0.7.
Threshold = 0.6

```
Epitopes   : ............................................EEEEEEEEEEEEEEEEEE.EEEEEEEEE......
Predictions: MDCCTENACSKPDDDILDIPLDDPGANAAAAKIQASFRGHMARKKIKSGERGRKGPGPGGPGGAGVARGGAGGGPSGD
             1--------10--------20--------30--------40--------50--------60--------70-------
```

ElliPro (Prediction) [48]

The two highest scoring epitope predictions both lie in the disordered region towards the C-terminus.

Score = 0.803

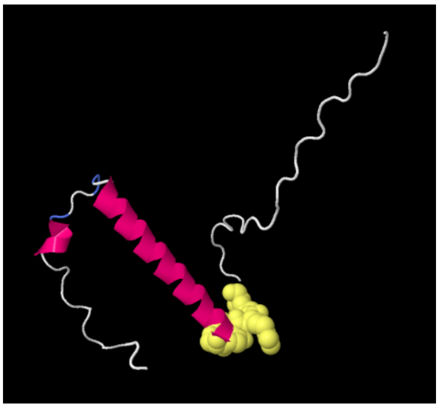

Score = 0.754

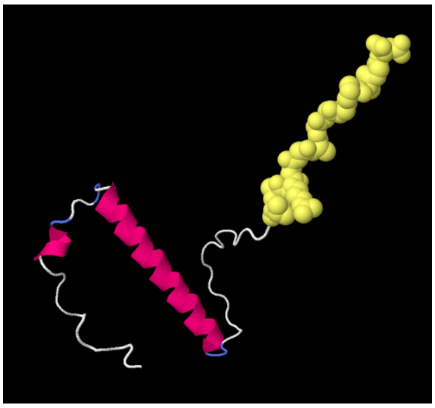

Interpretation
The epitope prediction agrees with each other and information collected from other tools. The disordered C-terminal region is the most suitable as an epitope.
Structure-based predictions in disordered regions should be evaluated carefully as these methods were trained on proteins with a secondary structure.

Known epitopes

IEDB (Database of annotations) [49]

No entries

SAbDab (Database of annotations) [50]

No entries

Interpretation
No information available on known epitopes of neurogranin.

Epitope specificity

BLAST (Calculation) [51]

There is some overlap between the amino acid sequence of neurogranin (pink) and other proteins especially within the central alpha helix region. Out of the three matches within the human proteome (neuromodulin, SPEG neighbour protein and Sperm protein 17) neuromodulin is the most concerning as it also contains the IQ domain present in neurogranin.

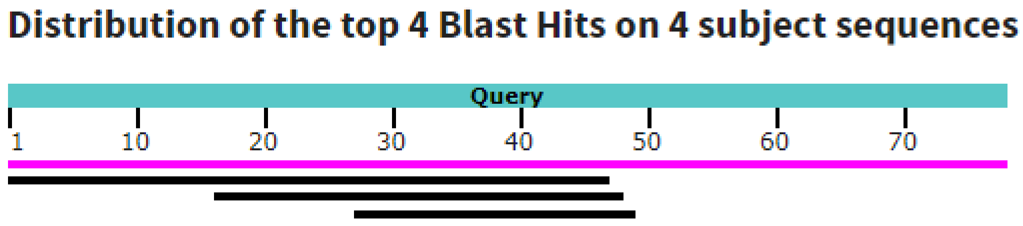

Interpretation

Cross-reactivity of anti-neurogranin antibodies with neuromodulin is possible if the epitope is located within the regions of higher sequence similarity. It should be checked if this has been tested by antibody suppliers.

Tau

Summary

Background

Tau is one of the most established and researched fluid dementia biomarkers. The concentration of total tau and phosphorylated tau are considered core CSF biomarkers for AD and are included as diagnosis criteria [52]. Tau has been shown to be highly predictive of cognitive decline in AD patients and its phosphorylated forms can be used for differential diagnosis from other dementia types such as FTD [53]. Much effort is currently put into implementing tau as a blood plasma biomarker as well [54].

Bioinformatics analysis

As tau is such an established AD biomarker, it is unsurprising that the analysis of its biological context strongly supports its potential as a fluid biomarker of dementia. Protein function, interaction partners and expression show a clear association to the brain and to AD specifically. The already existing knowledge on its disease involvement is extremely comprehensive but it is important to notice its involvement in other pathologies.

Further examination exposes various challenges or even obstacles to the successful immunobased detection of this protein. Tau has been found in extracellular vesicles, which might hinder detection of a subset of tau species present in plasma or CSF, its AlphaFold structure is predicted to contain several aggregation hot spots and is identified as an amyloid fibre-forming protein on AmyPro and the PDB. In addition, tau has a variety of proteoforms because of its many different splice variants and PTM sites.

In terms of structure, the predictions may be more difficult to interpret. The AlphaFold model of tau has a highly disordered, generally stretched out structure, with very little inter-residue contacts; there is only one helical region. This agrees with previous studies aiming to characterize the structure of tau [55], where it is indeed found that tau in its soluble form is not compactly folded, but an ensemble of different configurations with

transient secondary structures. In its native form, tau forms a molten-globule-like state; it is therefore more difficult to predict which residues are available as binding surfaces for an antibody. The results of the sequence-based structural predictors, NetSurfP-2.0 and DescribePROT do not agree with each other.

Reflection
Despite all these challenges, tau is well-established and researched as an immunoassay target and many immunogenic regions are known. Tau as a biomarker affirms that often identified points of caution or even obstacles can be handled or even utilized within the implementation of immunoassays. The existence of phosphorylation site and splicing specific forms of tau is firmly established and has been successfully exploited to develop more sensitive assays for specific tau forms [53, 56]. Measuring tau in EVs as a biomarker is also actively researched nowadays [57]. The information contained in bioinformatics tools and data resources is an obvious approach to identify novel tau proteoforms as biomarker candidates. The analysis identified tau as an aggregation protein which is known to be one of the hallmarks of AD pathogenesis [58].

Protein function

UniProt (Database of annotations) [8, 59]
GO - Molecular function (selection):

- actin binding
- apolipoprotein binding
- chaperone binding
- DNA binding

GO - Biological process (selection):

- activation of cysteine-type endopeptidase activity involved in apoptotic process
- amyloid fibril formation
- astrocyte activation
- axonal transport

Interpretation
The function of tau in the brain is established, its malfunction leading to neuronal death (as is the case in dementia) is therefore expected.

Interaction partners

STRING (Database of annotations, database of predictions) [10, 60]

- Glycogen synthase kinase-3 beta (GSK3B)
- Apolipoprotein E (APOE)
- Tyrosine-protein kinase Fyn (FYN)
- Cyclin-dependent-like kinase 5 (CDK5)
- 14-3-3 protein zeta/delta (YWHAZ)
- Serine/threonine-protein kinase MARK2 (MARK2)
- S100B
- Caspase-3 (CASP3)
- Alpha-synuclein (SNCA)
- Tubulin beta-3 chain (TUBB3)

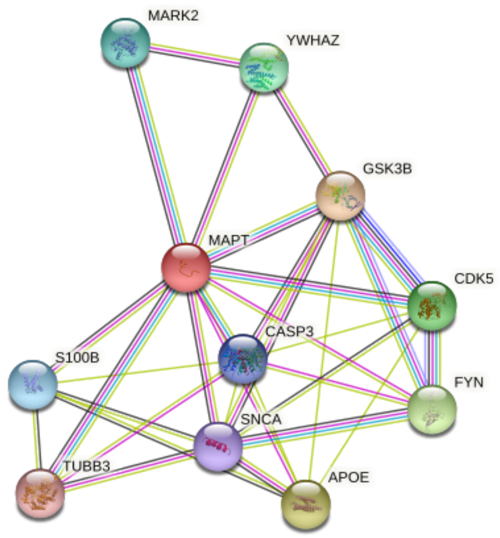

Interpretation
Tau known to interact with the AD risk factor APOE. It is also interacting with a protein involved in DLB pathology (SNCA). Its own role in dementia pathology is thus also conceivable.

Disease involvement

DisGeNET (Database of annotations) [12, 61]
Tau's strongest gene-disease association scores are connected to frontotemporal dementia (460 publications), AD (997 publications), Parkinsonian Disorders (101 publications). Tau is obviously well studied as a biomarker.

Interpretation
Tau is strongly established as a biomarker for dementia.
One should be aware of tau's involvement in several neurodegenerative diseases as it might not be a specific biomarker and not useful for differential diagnosis.

Tissue-specific expression

HPA (Database of annotations) [14, 62]
Tau is group enriched in the brain and skeletal muscle, but detected in many tissues.

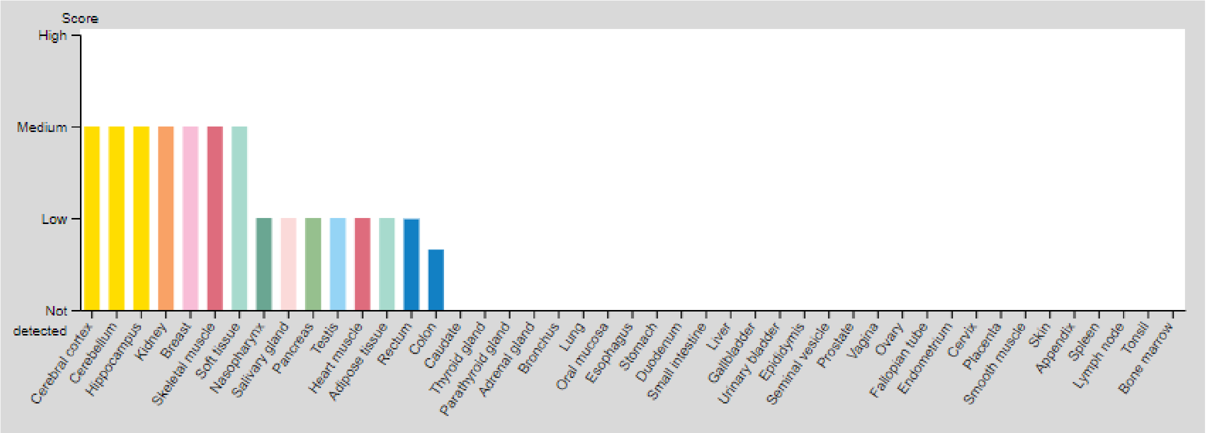

Image credit: Human Protein Atlas

HBFP (Database of annotations) [16]
Body fluids in which tau was identified:

- cerebrospinal fluid (13 papers in total) | confidence score: 0.945
- plasma/serum (41 papers in total) | confidence score: 0.81

UniProt (Database of annotations) [8, 59]
"Expressed in neurons. Isoform PNS-tau is expressed in the peripheral nervous system while the others are expressed in the central nervous system."

Interpretation
Tau shows enriched expression in the brain, tau detected in body fluids is thus likely to be of brain origin.
It is detected in other tissues as well, and the contribution of each tissue expression to the tau concentration in CSF and plasma is not clear.

Subcellular localization

UniProt (Database of annotations) [8, 59]
GO - Cellular component (selection):

- cytoskeleton
- secreted
- plasma membrane
- cytosol
- axon
- dendrite

Interpretation
The secretion of tau makes its detection in body fluids more likely. Its localization in brain cells (neurons) is established and supports its role in the nervous system.

EV localization

Vesiclepedia (Database of annotations) [17, 63]
Tau was identified in EVs in 5 different studies.

Interpretation
The presence of tau in EVs should be considered as it would be an obstacle to antibody detection.

Protein structure

Compute pI/Mw (Calculation) [19]
78.796 kDa

PDB (Database of annotations) [20, 21, 64, 65, 66]
Solved protein structures of tau with the highest coverage across its sequence are aggregated tau filaments (PDB ID: 6TJX), i.e., a pathogenic, not native fold.

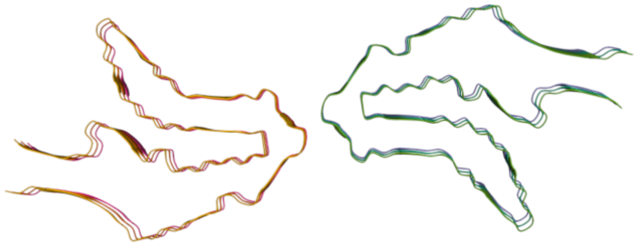

Several short stretches of amino acids are solved in which tau is in complex with other molecules such as microtubules and F-actin (PDB IDs: 2MZ7, 5NVB).

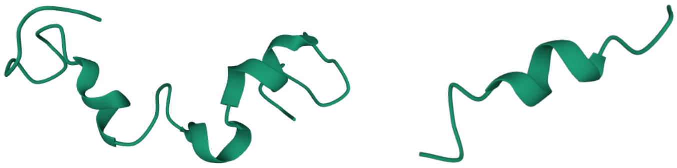

AlphaFold Protein Structure Database (Database of predictions) [23, 67]
AlphaFold predicts an almost exclusively disordered protein. The confidence is very low as structure prediction of disordered proteins is not feasible. Because of its size and high disorder content it is impossible to clearly identify which regions would be buried and which are accessible for antibodies.

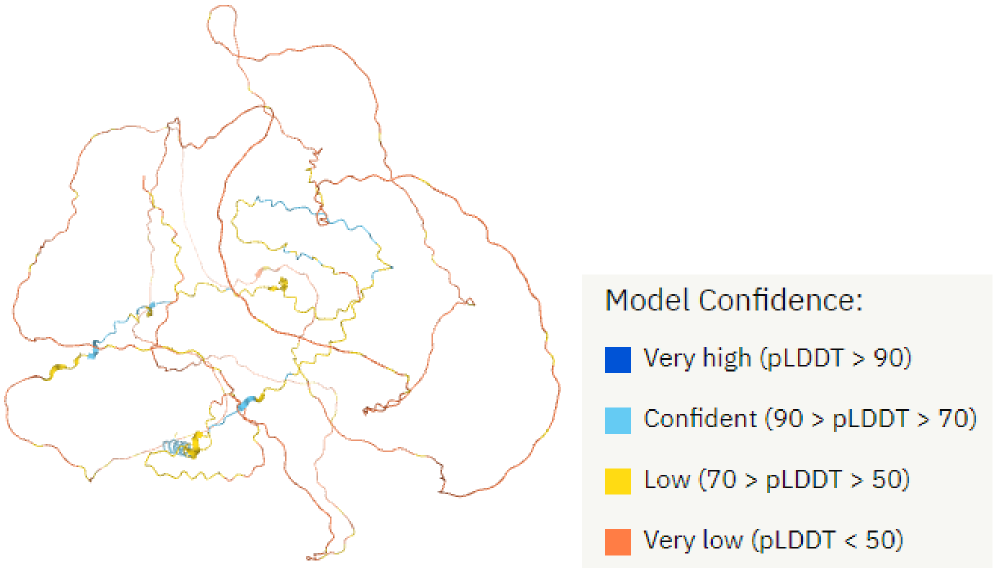

DescribePROT (Database of predictions) [25, 26, 27]
There is alternating prediction of coils, helices and strands. Many stretches are predicted as buried and would be inaccessible to antibodies.

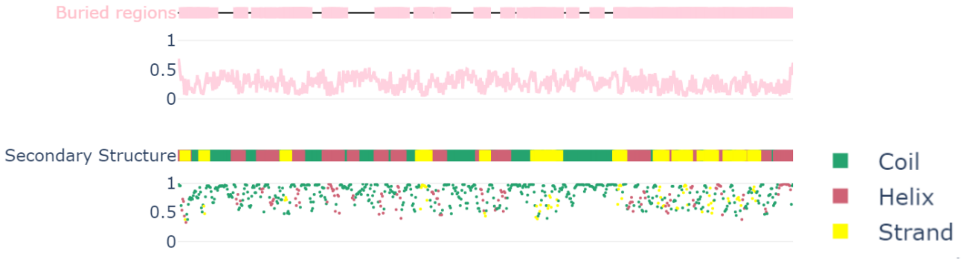

NetSurfP-2.0 (Prediction) [28]

All residues are predicted as disordered and surface accessible, no secondary structures are present at all in the structure model.

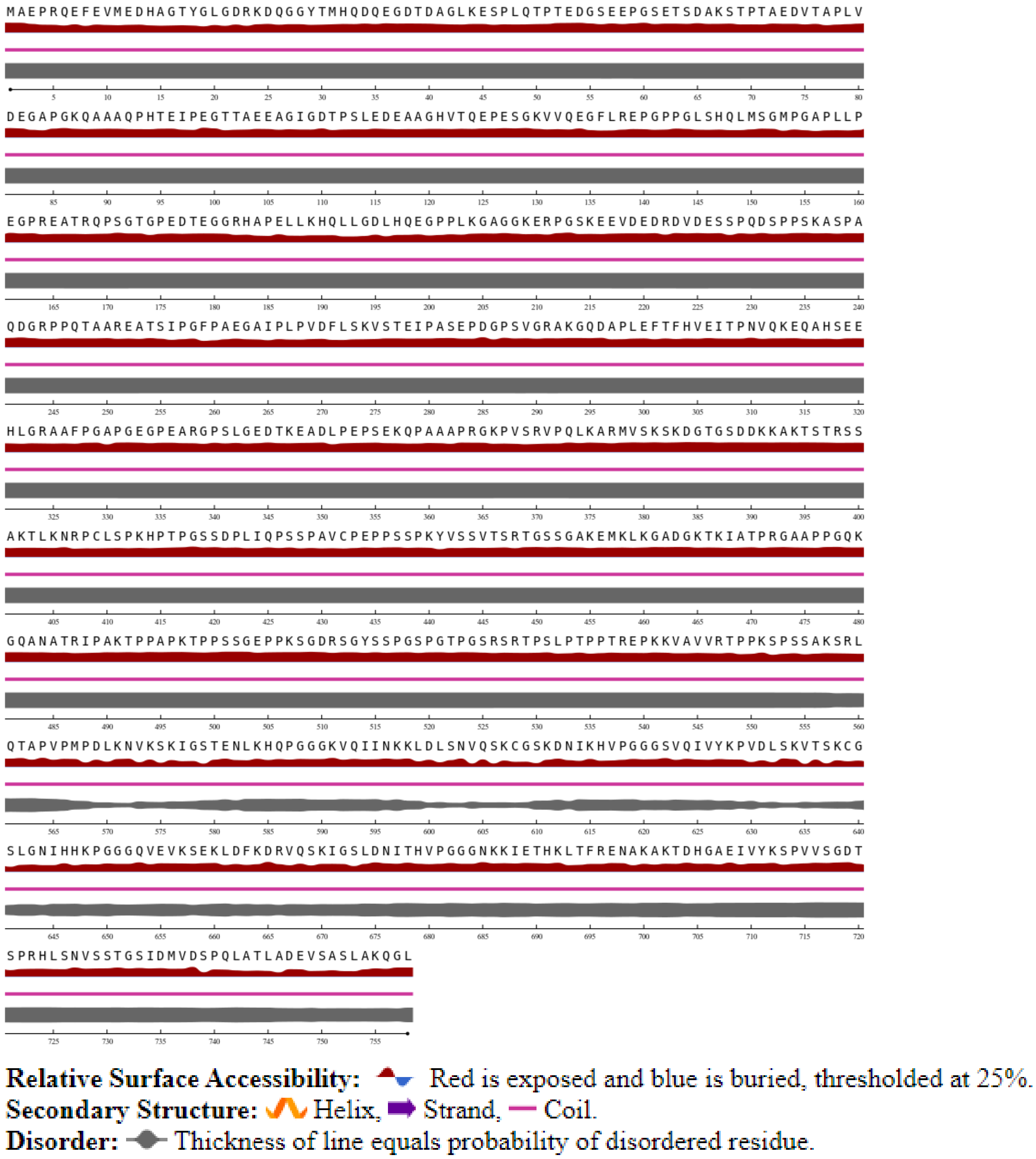

DisProt (Database of annotations) [29, 68]

Isoform Tau-F (441 amino acids) is entirely disordered and can create amyloid fibrils.

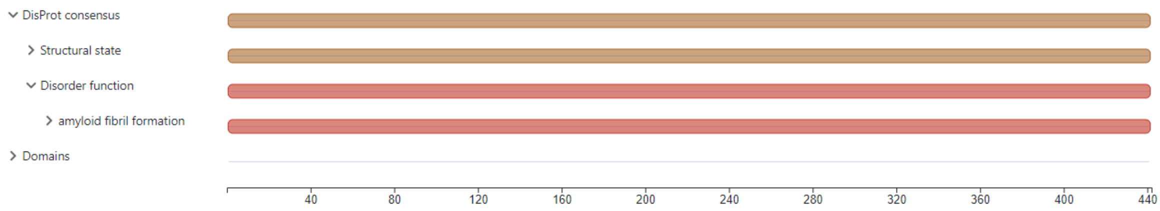

MobiDB (Database of annotations, database of predictions) [30, 69]
Disorder is predicted almost across the entire protein sequence of tau, propensity for secondary structures increases at C-terminus, which is also the interaction region of tau (i.e., the tubulin-binding repeat).

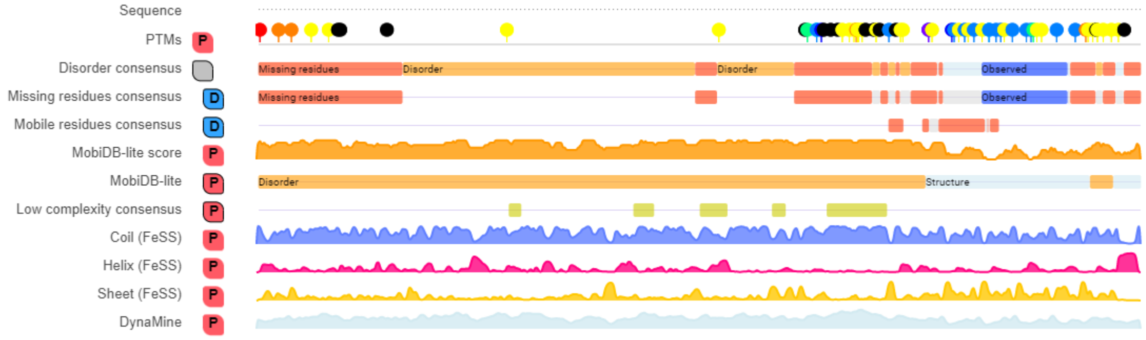

Interpretation
Tau is a large protein with high disorder content and thus offers a large surface area. An experimentally solved structure or a high confidence prediction are not available as tau is an intrinsically disordered protein that does not take a defined structure. The use of structural feature tools is thus limited. The predictions of DescribePROT do not agree with AlphaFold and NetSurfP-2.0.
Many residues might be not accessible for binding and it is very difficult to determine with confidence which residues are buried.

Isoforms and cleavage products

UniProt (Database of annotations) [8, 59]
"This entry describes 9 isoforms produced by alternative splicing. Note: Additional isoforms seem to exist. Isoforms differ from each other by the presence or absence of up to 5 of the 15 exons. One of these optional exons contains the additional tau/MAP repeat."

Interpretation
Many isoforms exist and some might not be identified yet. Antibodies might be only specific to some isoforms and thus would not give a full picture of tau concentrations.

PTMs

PhosphoSitePlus (Database of annotations) [32, 70]
Many phosphorylation annotations exist with high literature coverage.

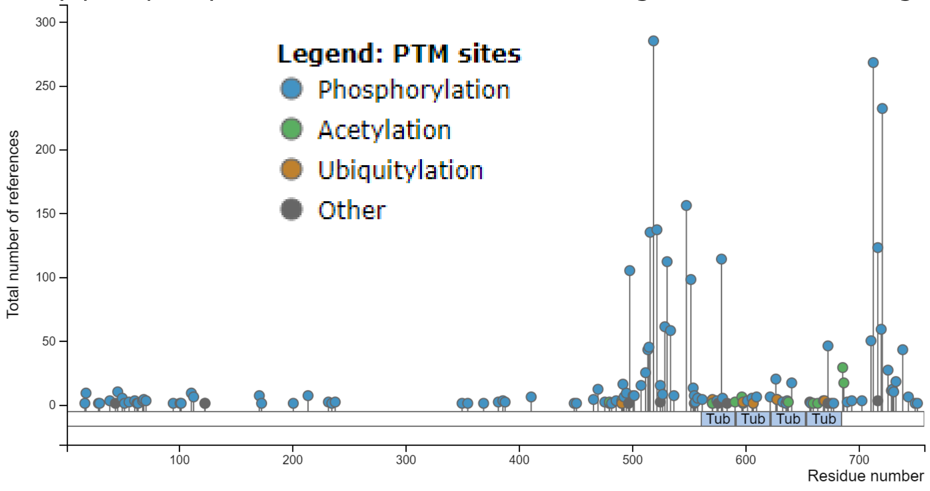

iPTMnet (Database of annotations) [34, 71]

Many phosphorylation annotations are annotated and the PTM sites differ between the different tau isoforms.

Interpretation
Many PTM sites exist across the entire sequence of tau and could hinder antibody-binding. It is difficult to evaluate how strong the hindrance of other closely positioned PTMs is on PTM-site specific antibodies.

Interaction residues

DescribePROT (Database of predictions) [25, 37, 38, 39]

The majority of the sequence is predicted as protein-binding. Barely no nucleotide-binding is predicted.

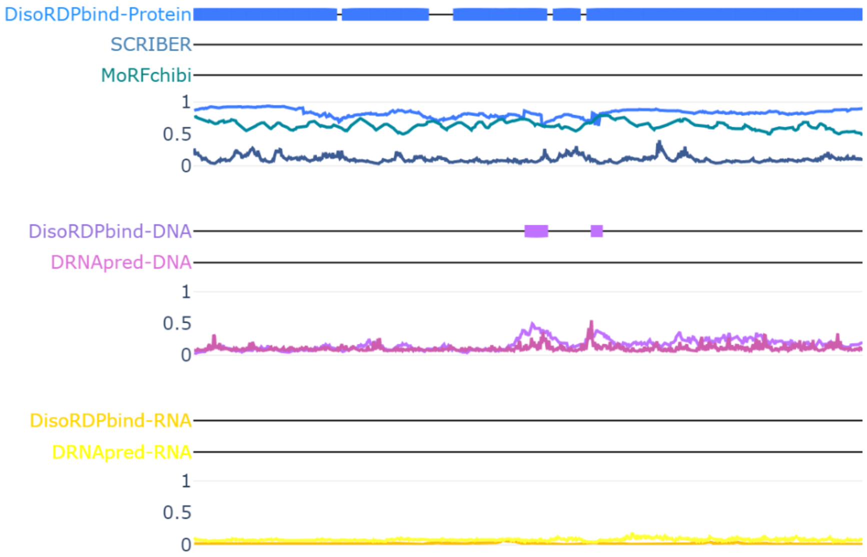

ANCHOR2 (Prediction) [40]

Disordered binding regions predicted with high propensity across almost the entire sequence. Only the C-terminus shows a decreasing disorder and disorder binding propensity.

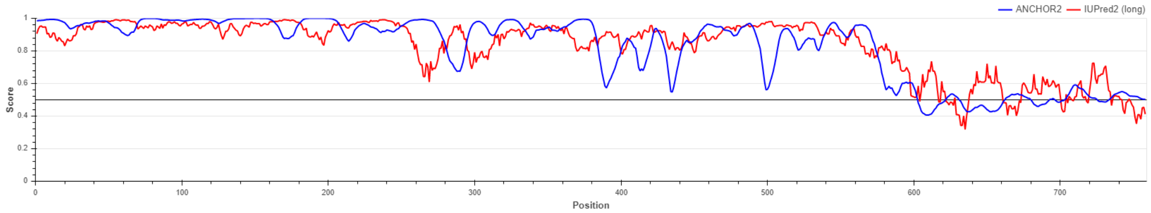

InterPRO (Database of annotations) [41, 72]

The tubulin-binding domains near the C-terminus of tau are known binding sites. As the relevant binding partners of tau are parts of the cellular cytoskeleton this is probably not of importance when detecting tau in fluids.

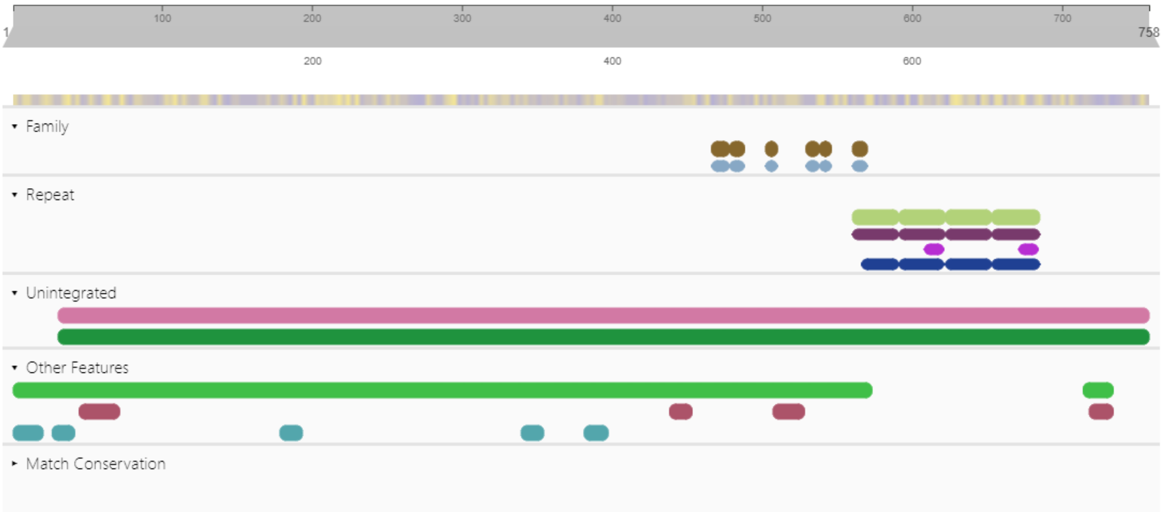

MobiDB (Database of annotations, database of predictions) [30, 69]
Linear interacting peptides are predicted across almost the entire protein sequence. Interactions are annotated at the C-terminus.

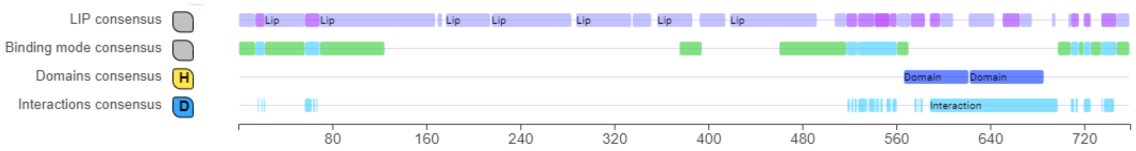

Interpretation
Predictions and annotations agree with each other and establish binding regions at the C-terminus and the potential to bind in most of the disordered regions of tau.
Protein binding could take place across most of the protein and hinder antibody binding.

Aggregation

Aggrescan3D 2.0 (Prediction) [43]
Several aggregation hotspots are predicted on the protein surface.

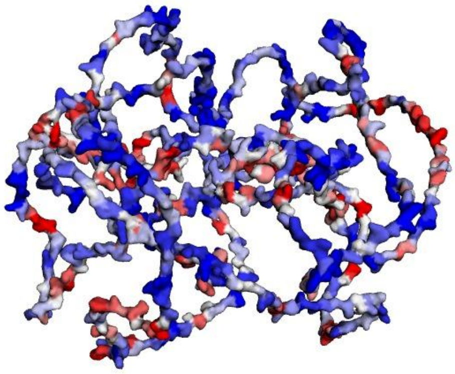

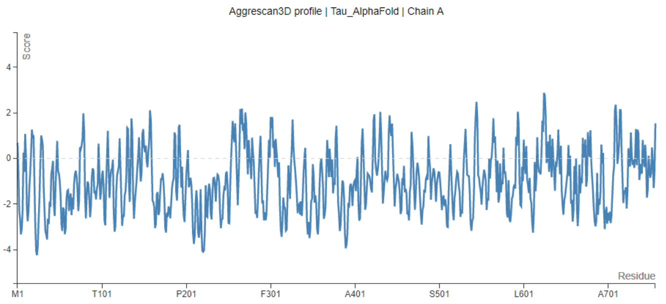

PASTA 2.0 (Prediction) [44]
The aggregation propensity is generally low across the entire sequence.

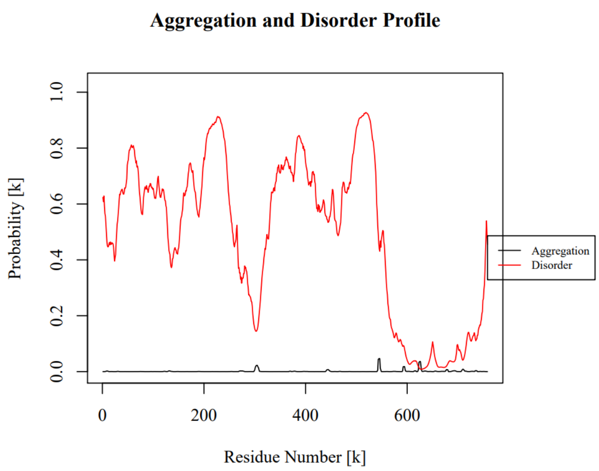

AmyPro (Database of annotations) [45, 73]

Tau contains a known pathogenic amyloid.

Investigated protein sequence (residues 1 to 441 of UniProt sequence P10636-8) :

MAEPRQEFEVMEDHAGTYGLGDRKDQGGYTMHQDQEGDTDAGLKESPLQTPTEDGSEEPGSETSDAKSTPTAEDVTAPLVDEGAPGKQAAAQPHTEIPEGTTAEEAGIGDTPSLEDEAAGHVTQARMVSKSKDGTGSDDK
KAKGADGKTKIATPRGAAPPGQKGQANATRIPAKTPPAPKTPPSSGEPPKSGDRSGYSSPGSPGTPGSRSRTPSLPTPPTREPKKVAVVRTPPKSPSSAKSRLQTAPVPMPDLKNVKSKIGSTENLKHQPGGGK**VQII**
NKKLDLSNVQSKCGSKDNIKHVPGGGS**VQIVYKPVDLSKVTSKCGSLGNIHHKPGGGQVEVKSEKLDFKDRVQSKIGSLDNITHVPGGG**
NKKIETHKLTFRENAKAKTDHGAEIVYKSPVVSGDTSPRHLSNVSSTGSIDMVDSPQLATLADEVSASLAKQGL

Interpretation

Tau is a known amyloid and contains several predicted aggregation hotspots. This could hamper antibody binding.

The structure- and sequence-based predictors do not agree with each other. It is important to remember that the structure-based predictions are based on the AlphaFold structure model which is not highly confident.

Epitope prediction

BepiPred-2.0 (Prediction) [47]

Practically the entire sequence of tau is predicted as an epitope at threshold 0.5. At a 0.6 threshold some gaps arise but still ~80% of residues are predicted as epitopes. No residue has a probability higher than 0.7 to be an epitope. All residues are predicted as exposed except for some residues at C-terminus.

ElliPro (Prediction) [48]

Many possible epitopes are predicted; the two highest ranking ones are shown below. Epitopes are mainly linear as they bind to disordered unfolded sequence stretches. The epitope ranked fourth (score = 0.83) contains the known PTM site at position 181 against which PTM-specific antibodies have been successfully raised.

Score = 0.89

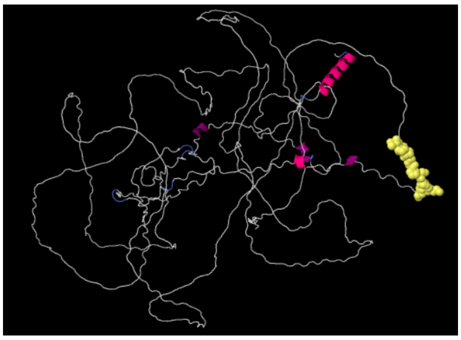

Score = 0.875

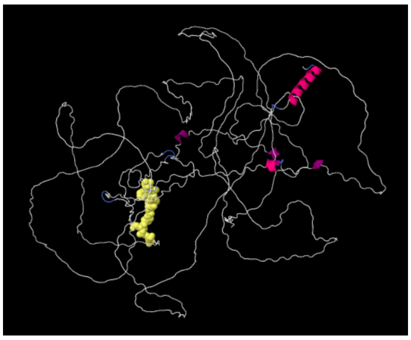

Interpretation
Vast stretches of tau are predicted as potential epitopes. There is not much distinction as it is an intrinsically disordered protein. Structure-based predictions in disordered regions should be evaluated carefully as methods were not trained on intrinsically disordered proteins. PTM sites cannot be included for epitope predictions.

Known epitopes

IEDB (Database of annotations) [49]

22 entries found of known tau epitopes.

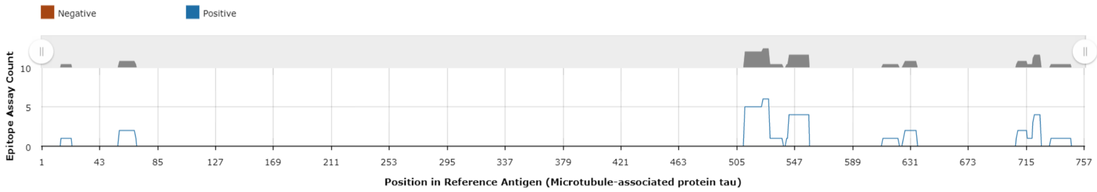

SAbDab (Database of annotations) [50]

7 entries found, all are tau peptides.

Interpretation
The information of epitope databases confirms that tau is immunogenic enough to raise antibodies at several locations.

Epitope specificity

BLAST (Calculation) [51]

Overlap with other microtubule-associated proteins 2 and 4, as well as Transmembrane protein PMIS2.

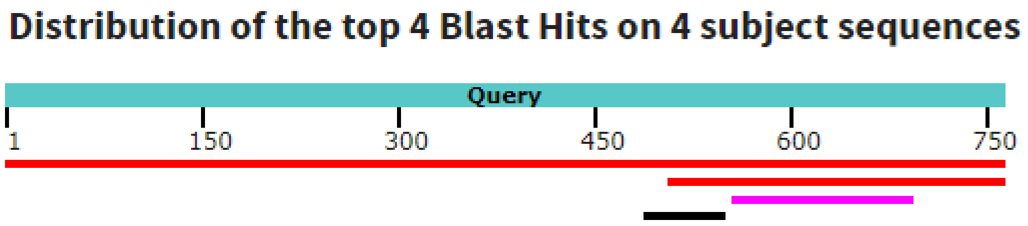

Interpretation
Cross-reactivity with other microtubule-associated proteins (both found in CSF and blood) is a potential problem.
This analysis should be performed isoform-specific as the potential cross-reactivity of anti-tau antibodies might strongly depend on the isoform to be detected.

TREM2

Summary

Background

TREM2 is a transmembrane protein expressed on microglia that has been suggested as a potential AD CSF biomarker [74]. Through proteolytic cleavage a soluble fragment (sTREM2) can be produced which is detectable in body fluids [75].

Bioinformatics analysis

Biological knowledge about TREM2 highlights its potential as a biomarker for dementia. It is clearly connected to AD and the associated proteins (e.g., amyloid-beta and APOE) through its function annotation and interaction partners. Additionally, it is known to be present in CSF. Compared to the other reviewed biomarkers the protein is much less specific to the brain. Moreover, involvement with other diseases might also limit its specificity as an AD biomarker.

Parts of the protein's structure are available in the PDB. While the AlphaFold structure model predicts those known domains of the protein well and with high confidence, the remaining regions of the sequence show less certainty and agreement with the sequence-based structure predictions. The prediction produced by Aggrescan3D 2.0 shows high aggregation propensity for the transmembrane helix region.

Much of the sequence of sTREM2 is part of the Ig-like domain. As this domain might be involved in binding an interaction partner and is not unique to the TREM2 protein, it does not constitute a good epitope for a TREM2-specific antibody. Considering the AlphaFold model and the NetSurfP-2.0 prediction for sTREM2, the C-terminal region of sTREM2 seems to be a suitable region for antibody binding. Because of the high disorder in this area, inaccessible residues are unlikely. Moreover, no PTM sites or binding sites are predicted there. This conclusion is supported by the BepiPred-2.0 epitope predictions with the highest confidence in the same region.

Reflection

Several predictions for TREM2 have to be considered carefully. While AlphaFold models can provide high confidence predictions on local secondary structures within a protein, the accuracy for inter-domain positions (e.g. in this case between the transmembrane helix and the extracellular domain) is less in some cases. Examining the TREM2 structure model by AlphaFold, provides a clear example of the limitations of these structure predictions for regions that are not compact but disordered as the relative position of the two domains of TREM2 is not possible. While the localization of the alpha helix in the plasma membrane is known to researchers, this knowledge is not explicitly factored in by machine learning predictors like AlphaFold and it can lead to wrong relative positioning of domains to each other. The existence of the transmembrane helix also affected the aggregation prediction. As aggregation prediction is largely based on the hydrophobicity of surface residues, the region of highest aggregation propensity corresponds to the highly hydrophobic transmembrane region. However, this region is incorporated into the membrane layer and not present in the soluble form; thus, it does not pose a challenge for sTREM2 detection in body fluids. The bioinformatics analysis of TREM2 exemplifies that information from prediction tools should always be scrutinized by including experimental knowledge during the interpretation. However, valuable information can still be derived from the presented resources regarding antibody or immunogenic peptide choice.

Protein function

UniProt (Database of annotations) [8, 76]

"Forms a receptor signaling complex with TYROBP which mediates signaling and cell activation following ligand binding."

GO - Molecular function (selection):

- amyloid-beta binding
- apolipoprotein A-I binding
- apolipoprotein binding
- high-density lipoprotein particle binding

GO - Biological process (selection):

- amyloid-beta clearance
- apoptotic cell clearance
- astrocyte activation
- cellular response to amyloid-beta

Interpretation

The association of TREM2 with the AD biomarker (amyloid-beta) strengthens its potential as a biomarker, especially as it is involved in amyloid-beta clearance, a process that is disturbed in AD pathology.

Interaction partners

STRING (Database of annotations, database of predictions) [10, 77]

- TYRO protein tyrosine kinase-binding protein (TYROBP)
- Plexin-A1(PLXNA1)
- Apolipoprotein E (APOE)
- 60 kDa heat shock protein, mitochondrial (HSPD1)
- Triggering receptor expressed on myeloid cells 1 (TREM1)
- Tyrosine-protein kinase SYK (SYK)
- Clusterin (CLU)
- Macrophage colony-stimulating factor 1 receptor (CSF1R)
- Tyrosine-protein phosphatase non-receptor type substrate 1 (SIRPA)
- Trem-like transcript 2 protein (TREML2)

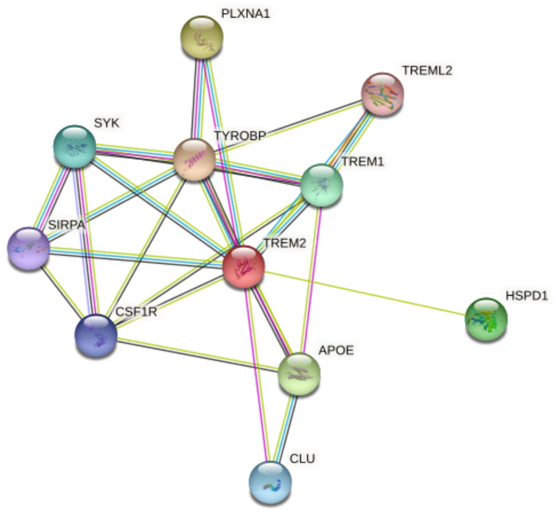

Interpretation

Interaction with the risk factor APOE strengthens the biomarker potential of TREM2.

Disease involvement

DisGeNET (Database of annotations) [12, 78]

Strongest gene-disease association scores are found for AD (214 publications), dementia (19 publications) and FTD (25 publications).

> **Interpretation**
> TREM2 is already considered as a biomarker for dementia.
> One should be aware of TREM2's involvement in several neurodegenerative diseases as it might not be a specific biomarker.

Tissue-specific expression

HPA (Database of annotations) [14, 79]

TREM2 is tissue enhanced in adipose tissue, brain, and lung, and detected in many additional tissues. It is less specific to the brain than the other biomarkers.

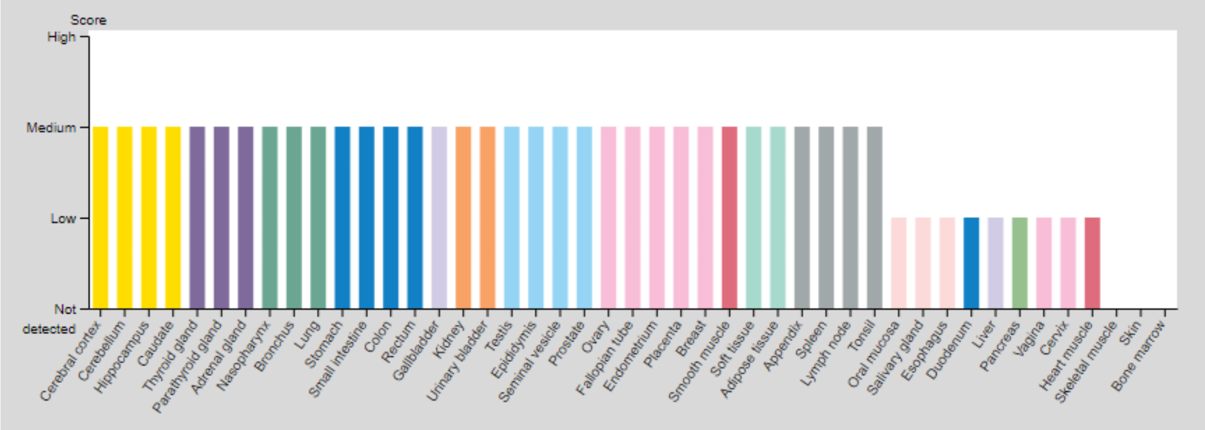

Image credit: Human Protein Atlas

HBFP (Database of annotations) [16]

Body fluids in which TREM2 was identified:

- cerebrospinal fluid (13 papers in total) | confidence score: 0.94

> **Interpretation**
> The expression of TREM2 is less specific, the origin of TREM2 in body fluids might be less certain.
> The highest expression is still found in brain tissue and its CSF presence is confirmed.

Subcellular localization

UniProt (Database of annotations) [8, 76]

- Isoform 1: Cell membrane
- Isoform 2: Secreted
- Isoform 3: Secreted

> **Interpretation**
> Two isoforms are secreted, the detection might be limited to these two isoforms in body fluids.

EV localization

Vesiclepedia (Database of annotations) [17, 80]

TREM2 was identified in blood serum exosomes in one study.

Interpretation

There is no definite evidence for or against EV localization of TREM2

Protein structure

Compute pI/Mw (Calculation) [19]

Full-length protein: 23.449 kDa

Extracellular domain (sTREM2): 17.434 kDa

PDB (Database of annotations) [20, 21, 81, 82]

Parts of the structure of TREM2 are experimentally solved and available: TREM2 extracellular domain (green, amino acids 19-131) in complex with a single-chain variable fragment (PDB ID: 6YYE) and TREM2 transmembrane helix (PDB ID: 6Z0I).

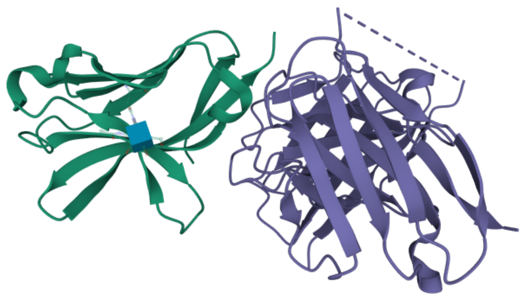

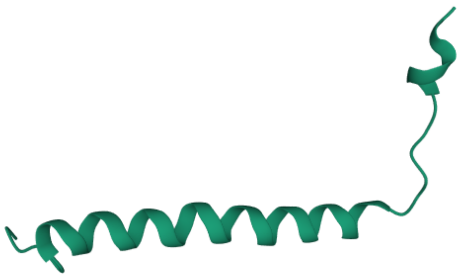

AlphaFold Protein Structure Database (Database of predictions) [23, 83]

The structurally known transmembrane alpha helix and beta sheets of the extracellular domain are predicted with confidence. Other regions are disordered with low confidence because of the missing stable structure. The relative localization of protein regions to each other cannot be correct as the alpha helix is embedded into the plasma membrane while the extracellular region is not attached to the membrane.

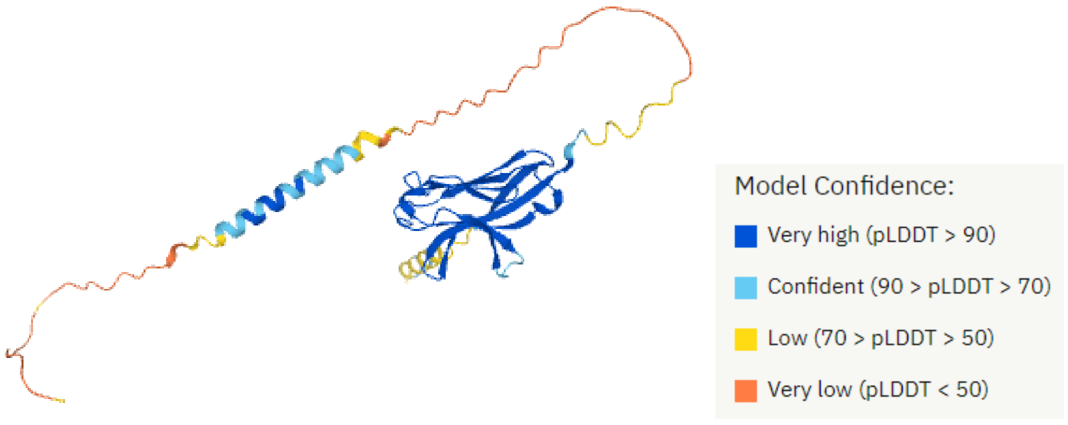

DescribePROT (Database of predictions) [25, 26, 27]

The sequence-based predictor correctly predicts the transmembrane alpha helix but disagrees with the AlphaFold model within the extracellular region (alternating helices and sheets instead of only sheets). It does not predict any long stretches of disordered/coil regions and many buried residues are predicted.

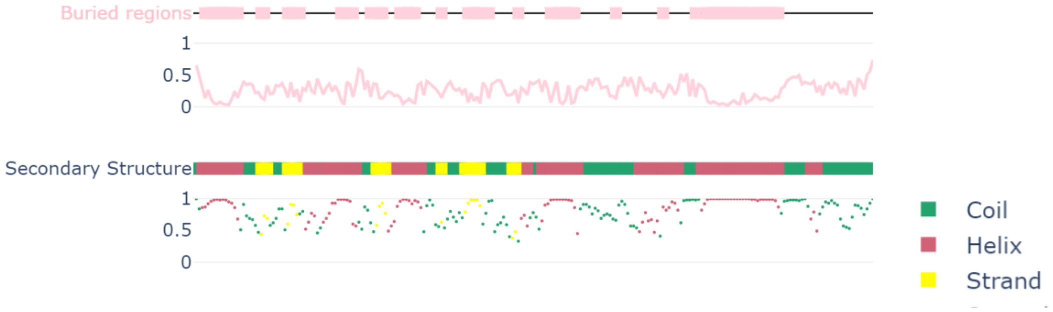

NetSurfP-2.0 (Prediction) [28]

The predictions of the long alpha helix and the interchanging beta sheets and disordered regions in the extracellular domain are similar to the AlphaFold model. The C-terminus of sTREM2 is predicted as a long disordered stretch.

Full-length protein (TREM2)

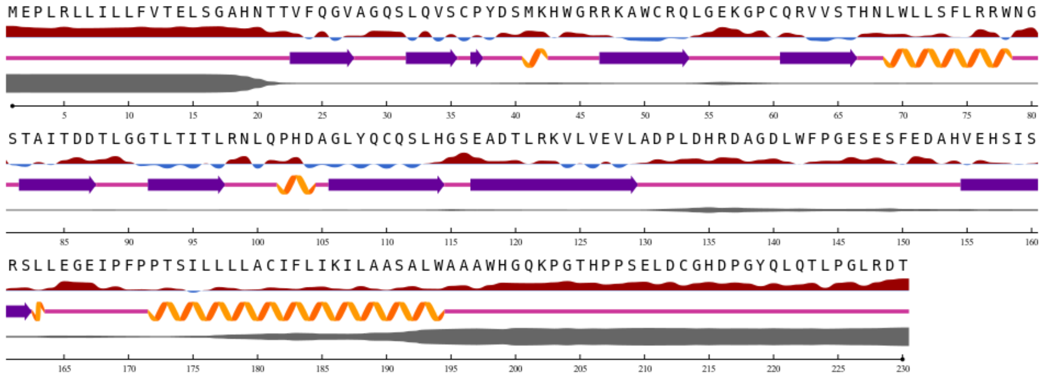

Extracellular domain (sTREM2)

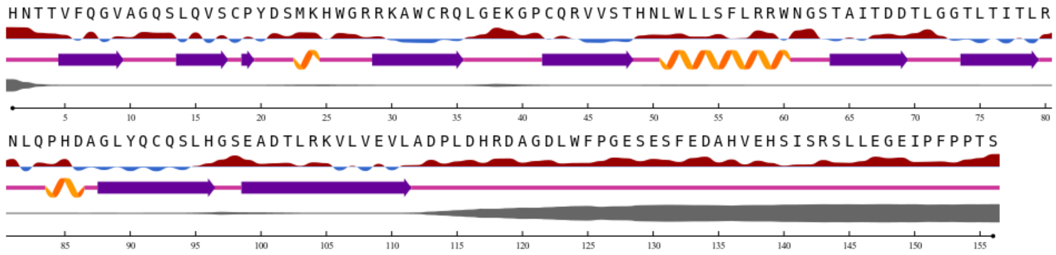

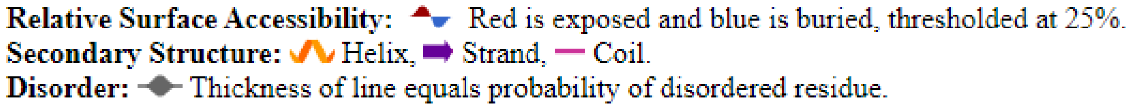

DisProt (Database of annotations) [29]

No entry found.

MobiDB (Database of annotations, database of predictions) [30, 84]

Most of the extracellular region is part of a domain. Alternating coils, helices and sheets are predicted with only the C-terminus (intracellular region) predicted as a likely disordered stretch.

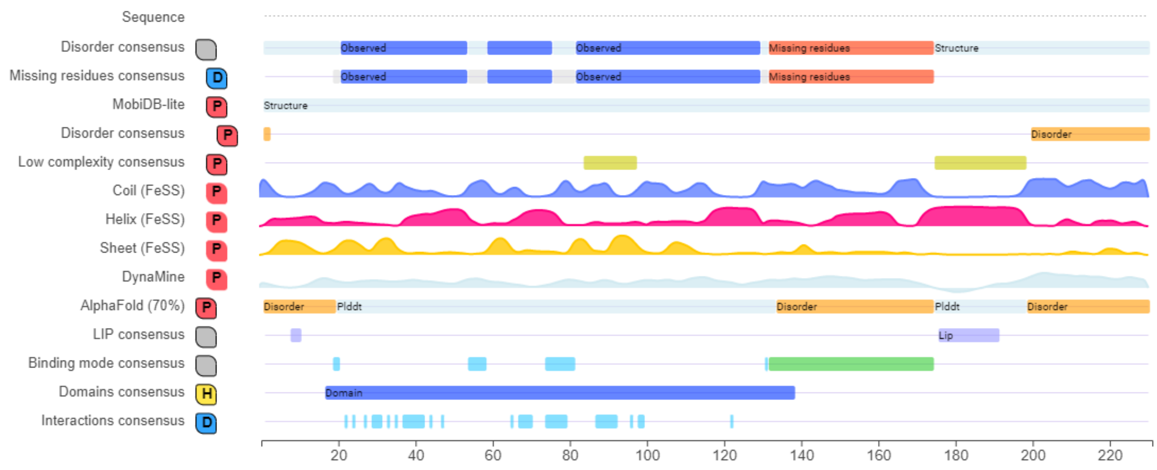

Interpretation
The structured regions of TREM2 (transmembrane and extracellular domain) have been solved and show high agreement with the AlphaFold predicted model.
Sequence-based predictors show less long stretches of disordered regions than the AlphaFold model. The structure of the extracellular region is less certain.
All models indicate that some residues are buried and might not be available for binding.

Isoforms and cleavage products

UniProt (Database of annotations) [8, 76]

- *"This entry describes 3 isoforms produced by alternative splicing."*
- *"Undergoes ectodomain shedding through proteolytic cleavage by ADAM10 and ADAM17 to produce a transmembrane segment, the TREM2 C-terminal fragment (TREM2-CTF), which is subsequently cleaved by gamma-secretase."*
- *"After ectodomain shedding, the extracellular domain oligomerizes, which is enhanced and stabilized by binding of phosphatidylserine."*

Interpretation
The isoform specificity of antibodies needs to be considered. Oligomerization of the extracellular domain could hinder antibody binding in body fluid samples.

PTMs

PhosphoSitePlus (Database of annotations) [32, 85]

Not many PTM sites are annotated and there is low coverage of associated references.

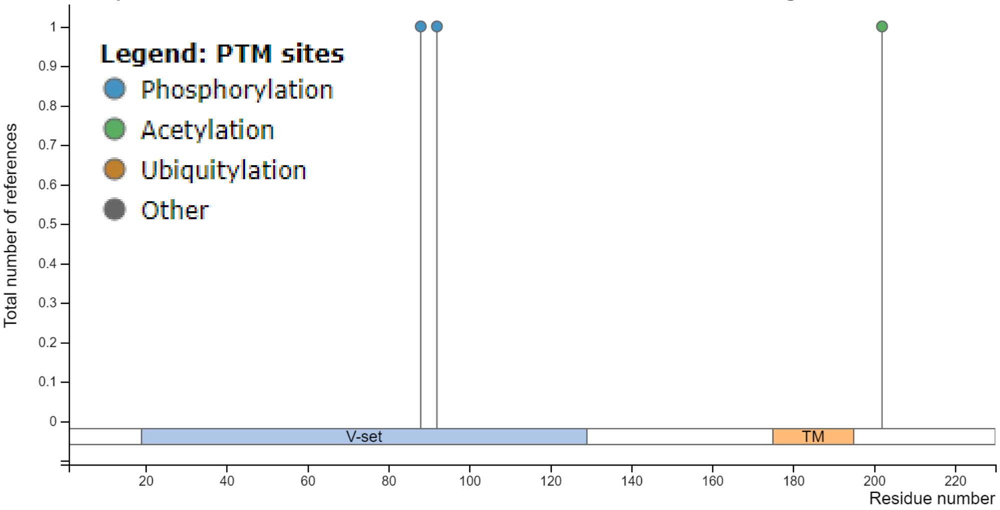

iPTMnet (Database of annotations) [34, 86]

Identical PTM sites are identified as by PhosphoSitePlus with generally low to medium confidence scores. An additional glycosylation site towards the N-terminus exists.

Site	PTM Type	PTM Enzyme	Score
All	All		All
N20	N-Glycosylation		★★☆☆
N79	N-Glycosylation		★★☆☆
T88	Phosphorylation		★☆☆☆
T92	Phosphorylation		★☆☆☆

MusiteDeep (Prediction) [36]

The PTM prediction partly overlaps with the annotations. Additional PTM sites are predicted towards the C-terminus of TREM2.

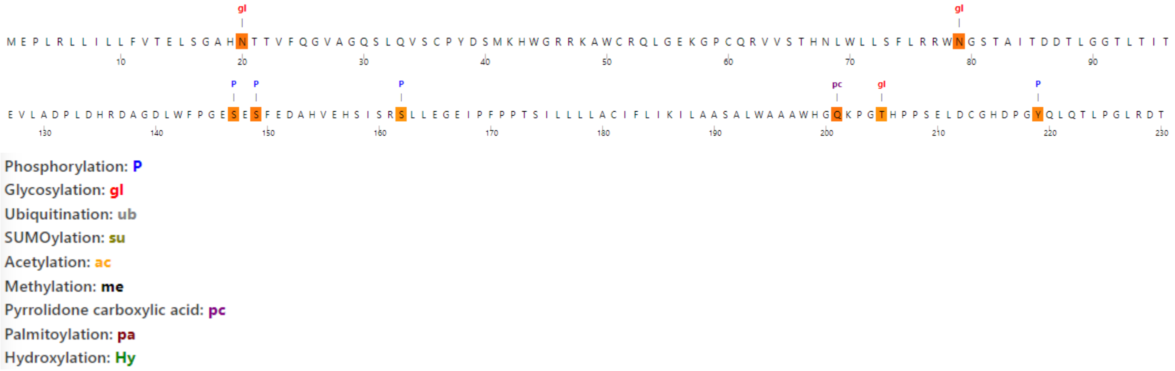

Interpretation

No PTM annotations are found between the V-set domain and the transmembrane region, thus it might be a suitable location for antibody binding.

The low coverage of PTM references indicates that TREM2 is not well studied yet. Predictions of additional PTM sites should be considered.

Interaction residues

DescribePROT (Database of predictions) [25, 37, 38, 39]

No protein or RNA binding and barely any DNA binding is predicted for TREM2.

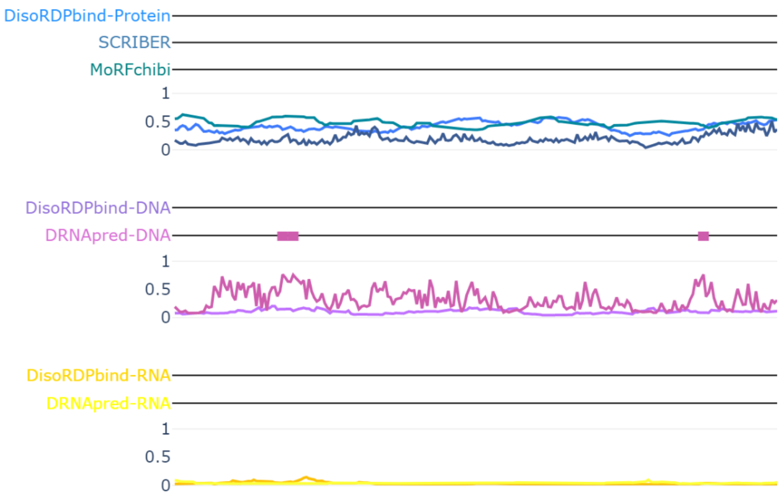

ANCHOR2 (Prediction) [40]

There is a high disorder prediction at the intracellular region. Generally, the predictor indicates a low probability of disordered binding regions.

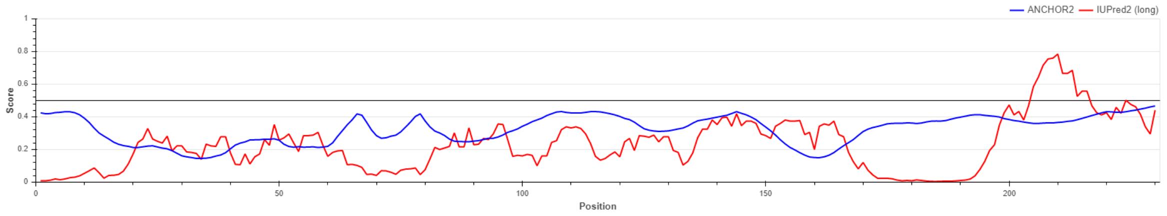

InterPRO (Database of annotations) [41, 87]

The folded part of the extracellular region is the Ig-like domain which is common across proteins.

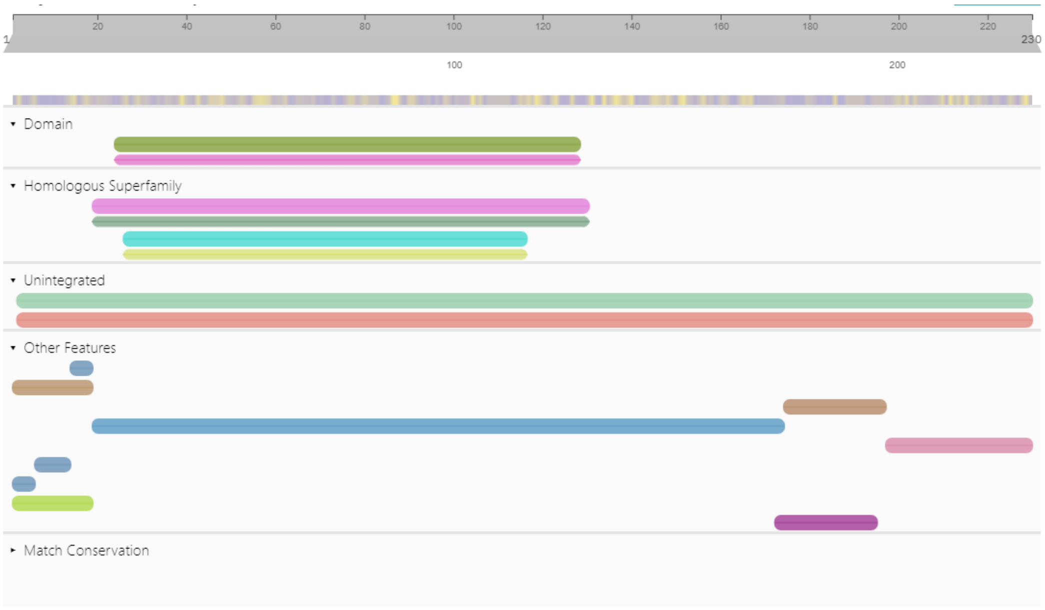

MobiDB (Database of annotations, database of predictions) [30, 84]
Disorder to disorder binding (green) is annotated close to the transmembrane region. Only small linear binding peptides are predicted, interaction annotations are mostly derived from interactions with the Ig-like domain.

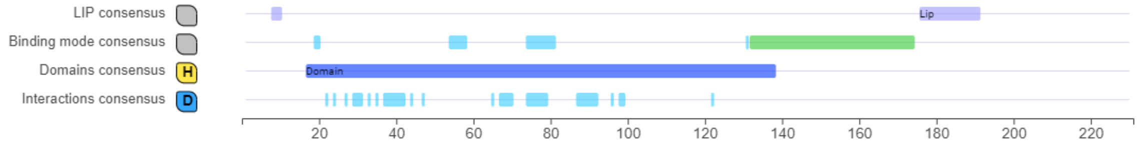

**Interpretation**<br>Low predicted binding propensity indicates that it is unlikely that TREM2 is interacting with other molecules.<br>A large part of the extracellular region is a domain which is likely to interact with other molecules and is not specific to TREM2.

Aggregation

Aggrescan3D 2.0 (Prediction) [43]
High aggregation propensity is predicted for the transmembrane helix and N-terminus of the extracellular region. The high aggregation propensity is caused by the hydrophobicity of transmembrane helix residues; however, aggregation will not occur as these residues are embedded into the plasma membrane and not exposed to solvent.

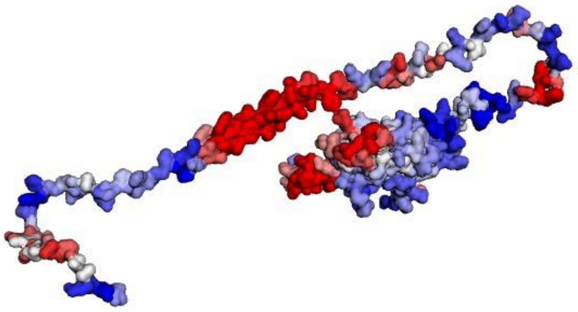

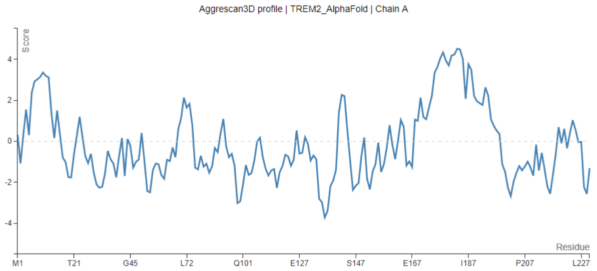

PASTA 2.0 (Prediction) [44]
A generally low score for aggregation propensity is found across the sequence. The highest aggregation propensity is around the transmembrane helix because of the high amount of hydrophobic residues.

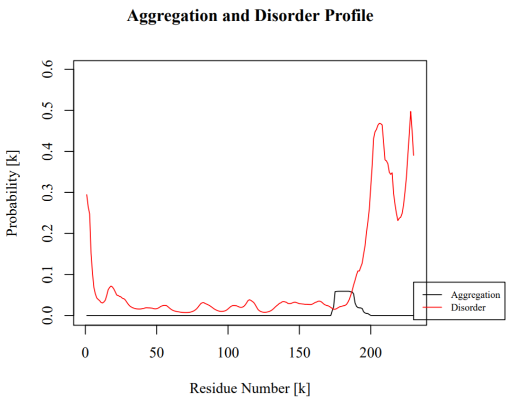

AmyPro (Database of annotations) [45]
No entry found.

Interpretation
The high aggregation propensity of Aggrescan shows the limitations of prediction models as the hydrophobicity of the transmembrane region is evaluated as an aggregation prone region despite its embedding into the plasma membrane. The transmembrane region is not part of the biomarker-relevant region, i.e., sTREM2.
Further hotspots are detected that might facilitate aggregation.

Epitope prediction

BepiPred-2.0 (Prediction) [47]
Several epitopes are identified for sTREM2 at threshold 0.5, at a higher threshold of 0.6 only one region between 135-165 amino acids is still predicted as an epitope.
Threshold=0.5

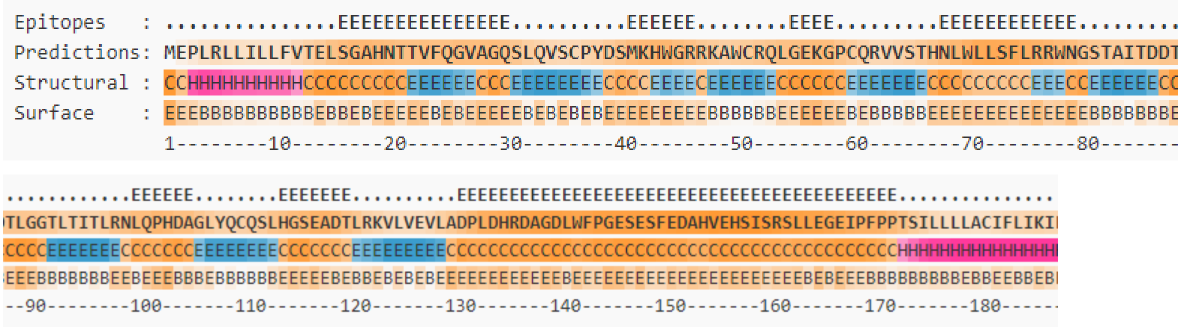

ElliPro (Prediction) [48]
The highest ranked epitope lies in the cytoplasmic region which is not relevant for sTREM2 detection. The other epitope is located in the extracellular region between the transmembrane helix and Ig-like domain.

Score = 0.83

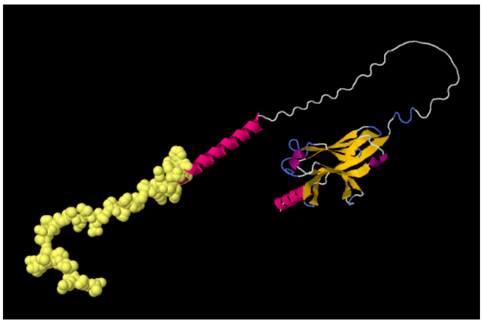

Score = 0.815

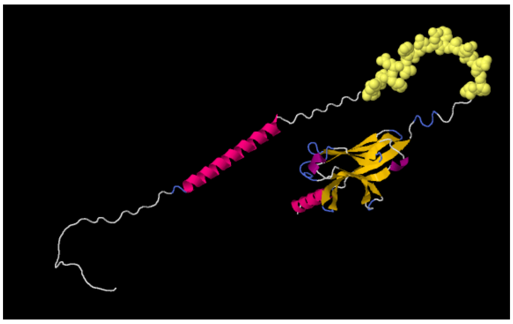

Interpretation
Epitope predictions agree with the other methods: the region close to the transmembrane helix on the extracellular site seems to constitute a good epitope.
Structure-based predictions in disordered regions should be evaluated carefully as methods were not trained on intrinsically disordered proteins.

Known epitopes

IEDB (Database of annotations) [49]
No entries found

SAbDab (Database of annotations) [50]
No results found

Interpretation
No information available on known epitopes of TREM2.

Epitope specificity

BLAST (Calculation) [51]

The Ig-like domain is not unique to TREM2. The proteins showing the highest alignment with TREM2 include Polymeric immunoglobulin receptor and Natural cytotoxicity triggering receptor 2.

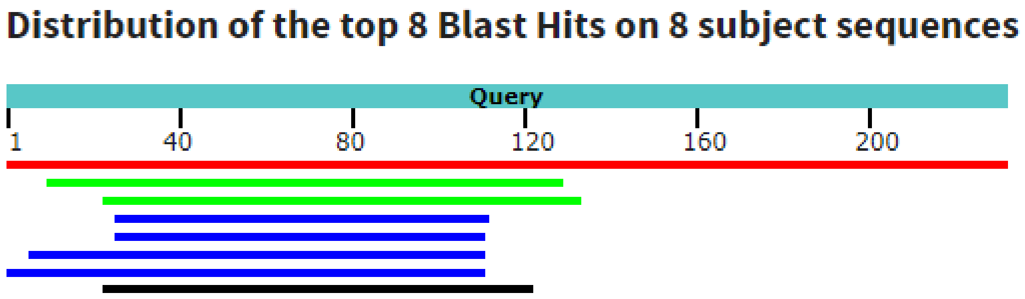

An epitope within the Ig-like domain could lead to cross-reactivity with other proteins carrying this domain and should therefore be avoided.

References

1. Nazir FH, Camporesi E, Brinkmalm G, Lashley T, Toomey CE, Kvartsberg H, et al. Molecular forms of neurogranin in cerebrospinal fluid. Journal of Neurochemistry. 2020 Dec 17;157(3):816–33.

2. Blennow K, Zetterberg H. Biomarkers for Alzheimer's disease: current status and prospects for the future. Journal of Internal Medicine. 2018 Aug 19;284(6):643–63.

3. Wellington H, Paterson RW, Portelius E, Törnqvist U, Magdalinou N, Fox NC, et al. Increased CSF neurogranin concentration is specific to Alzheimer disease. Neurology. 2016 Jan 29;86(9):829–35.

4. Portelius E, Zetterberg H, Skillbäck T, Törnqvist U, Andreasson U, Trojanowski JQ, et al. Cerebrospinal fluid neurogranin: relation to cognition and neurodegeneration in Alzheimer's disease. Brain. 2015 Sep 15;138(11):3373–85.

5. Kester MI, Teunissen CE, Crimmins DL, Herries EM, Ladenson JackH, Scheltens P, et al. Neurogranin as a Cerebrospinal Fluid Biomarker for Synaptic Loss in Symptomatic Alzheimer Disease. JAMA Neurology. 2015 Nov 1;72(11):1275.

6. De Vos A, Struyfs H, Jacobs D, Fransen E, Klewansky T, De Roeck E, et al. The Cerebrospinal Fluid Neurogranin/BACE1 Ratio is a Potential Correlate of Cognitive Decline in Alzheimer's Disease. Journal of Alzheimer's Disease. 2016 Aug 8;53(4):1523–38.

7. Willemse EAJ, De Vos A, Herries EM, Andreasson U, Engelborghs S, van der Flier WM, et al. Neurogranin as Cerebrospinal Fluid Biomarker for Alzheimer Disease: An Assay Comparison Study. Clinical Chemistry. 2018 Jun 1;64(6):927–37.

8. Bateman A, Martin M-J, Orchard S, Magrane M, Agivetova R, Ahmad S, et al. UniProt: the universal protein knowledgebase in 2021. Nucleic Acids Research. 2020 Nov 25;49(D1):D480–9.

9. NRGN - Neurogranin - Homo sapiens (Human). UniProt. https://www.uniprot.org/uniprot/Q92686. Accessed 07 February 2021.

10. Szklarczyk D, Gable AL, Lyon D, Junge A, Wyder S, Huerta-Cepas J, et al. STRING v11: protein–protein association networks with increased coverage, supporting functional discovery in genome-wide experimental datasets. Nucleic Acids Research. 2018 Nov 22;47(D1):D607–13.

11. NRGN protein (human) - STRING interaction network. STRING. https://version-11-5.string-db.org/cgi/network?networkId=brl9zwOE4CZJ. Accessed 07 February 2021.

12. Piñero J, Ramírez-Anguita JM, Saüch-Pitarch J, Ronzano F, Centeno E, Sanz F, et al. The DisGeNET knowledge platform for disease genomics: 2019 update. Nucleic Acids Research. 2019 Nov 4;

13. Gene-disease association data retrieved from DisGeNET v7.0. Integrative Biomedical Informatics Group GRIB/IMIM/UPF. DisGeNET. https://www.disgenet.org/browser/1/1/0/4900/. Accessed 07 February 2021.

14. Uhlén M, Fagerberg L, Hallström BM, Lindskog C, Oksvold P, Mardinoglu A, et al. Tissue-based map of the human proteome. Science. 2015 Jan 23;347(6220).

15. NRGN protein expression summary. The Human Protein Atlas. Available from: https://v21.proteinatlas.org/ENSG00000154146-NRGN. Accessed 07 February 2021.

16. Shao D, Huang L, Wang Y, Cui X, Li Y, Wang Y, et al. HBFP: a new repository for human body fluid proteome. Database. 2021 Oct 1;2021.

17. Pathan M, Fonseka P, Chitti SV, Kang T, Sanwlani R, Van Deun J, et al. Vesiclepedia 2019: a compendium of RNA, proteins, lipids and metabolites in extracellular vesicles. Nucleic Acids Research. 2018 Nov 5;47(D1):D516–9.

18. Gene summary - NRGN. Vesiclepedia. http://microvesicles.org/gene_summary?gene_id=4900. Accessed 07 February 2021.

19. Duvaud S, Gabella C, Lisacek F, Stockinger H, Ioannidis V, Durinx C. Expasy, the Swiss Bioinformatics Resource Portal, as designed by its users. Nucleic Acids Research. 2021 Apr 13;49(W1):W216–27.

20. Berman HM. The Protein Data Bank. Nucleic Acids Research. 2000 Jan 1;28(1):235–42.

21. Burley SK, Bhikadiya C, Bi C, Bittrich S, Chen L, Crichlow GV, et al. RCSB Protein Data Bank: powerful new tools for exploring 3D structures of biological macromolecules for basic and applied research and education in fundamental biology, biomedicine, biotechnology, bioengineering and energy sciences. Nucleic Acids Research. 2020 Nov 19;49(D1):D437–51.

22. Kumar V, Chichili VPR, Zhong L, Tang X, Velazquez-Campoy A, Sheu F-S, et al. Structural Basis for the Interaction of Unstructured Neuron Specific Substrates Neuromodulin and Neurogranin with Calmodulin. Scientific Reports. 2013 Mar 6;3(1).

23. Varadi M, Anyango S, Deshpande M, Nair S, Natassia C, Yordanova G, et al. AlphaFold Protein Structure Database: massively expanding the structural coverage of protein-sequence space with high-accuracy models. Nucleic Acids Research. 2021 Nov 17;50(D1):D439–44.

24. Zhao B, Katuwawala A, Oldfield CJ, Dunker AK, Faraggi E, Gsponer J, et al. DescribePROT: database of amino acid-level protein structure and function predictions. Nucleic Acids Research. 2020 Oct 29;49(D1):D298–308.

25. AlphaFold Protein Structure Database. https://alphafold.ebi.ac.uk/entry/Q92686. Accessed 07 February 2021.

26. Faraggi E, Kouza M, Zhou Y, Kloczkowski A. Fast and Accurate Accessible Surface Area Prediction Without a Sequence Profile. In: Methods in Molecular Biology. New York, NY: Springer New York; 2016. p. 127–36.

27. Jones DT. Protein secondary structure prediction based on position-specific scoring matrices. Journal of Molecular Biology. 1999 Sep;292(2):195–202.

28. Klausen MS, Jespersen MC, Nielsen H, Jensen KK, Jurtz VI, Sønderby CK, et al. NetSurfP-2.0: Improved prediction of protein structural features by integrated deep learning. Proteins: Structure, Function, and Bioinformatics. 2019 Mar 9;87(6):520–7.

29. Quaglia F, Mészáros B, Salladini E, Hatos A, Pancsa R, Chemes LB, et al. DisProt in 2022: improved quality and accessibility of protein intrinsic disorder annotation. Nucleic Acids Research. 2021 Nov 25;50(D1):D480–7.

30. Piovesan D, Necci M, Escobedo N, Monzon AM, Hatos A, Mičetić I, et al. MobiDB: intrinsically disordered proteins in 2021. Nucleic Acids Research. 2020 Nov 25;49(D1):D361–7.

31. MobiDB. https://mobidb.bio.unipd.it/Q92686. Accessed 07 February 2021.

32. Hornbeck PV, Kornhauser JM, Tkachev S, Zhang B, Skrzypek E, Murray B, et al. PhosphoSitePlus: a comprehensive resource for investigating the structure and function of experimentally determined post-translational modifications in man and mouse. Nucleic Acids Research. 2011 Dec 1;40(D1):D261–70.

33. Neurogranin (human). PhosphoSitePlus. https://www.phosphosite.org/proteinAction?id=7623&showAllSites=true. Accessed 07 February 2021.

34. Huang H, Arighi CN, Ross KE, Ren J, Li G, Chen S-C, et al. iPTMnet: an integrated resource for protein post-translational modification network discovery. Nucleic Acids Research. 2017 Nov 14;46(D1):D542–50.

35. iPTMnet Report Q92686 NRGN. iPTMnet. https://research.bioinformatics.udel.edu/iptmnet/entry/Q92686/. Accessed 07 February 2021.

36. Wang D, Liu D, Yuchi J, He F, Jiang Y, Cai S, et al. MusiteDeep: a deep-learning based webserver for protein post-translational modification site prediction and visualization. Nucleic Acids Research. 2020 Apr 23;48(W1):W140–6.

37. Peng Z, Kurgan L. High-throughput prediction of RNA, DNA and protein binding regions mediated by intrinsic disorder. Nucleic Acids Research. 2015 Jun 24;43(18):e121–e121.

38. Zhang J, Kurgan L. SCRIBER: accurate and partner type-specific prediction of protein-binding residues from proteins sequences. Bioinformatics. 2019 Jul;35(14):i343–53.

39. Malhis N, Jacobson M, Gsponer J. MoRFchibi SYSTEM: software tools for the identification of MoRFs in protein sequences. Nucleic Acids Research. 2016 May 12;44(W1):W488–93.

40. Erdős G, Dosztányi Z. Analyzing Protein Disorder with IUPred2A. Current Protocols in Bioinformatics. 2020 Apr;70(1).

41. Blum M, Chang H-Y, Chuguransky S, Grego T, Kandasaamy S, Mitchell A, et al. The InterPro protein families and domains database: 20 years on. Nucleic Acids Research. 2020 Nov 6;49(D1):D344–54.

42. InterPro. https://www.ebi.ac.uk/interpro/protein/UniProt/Q92686/. Accessed 07 February 2021.

43. Kuriata A, Iglesias V, Pujols J, Kurcinski M, Kmiecik S, Ventura S. Aggrescan3D (A3D) 2.0: prediction and engineering of protein solubility. Nucleic Acids Research. 2019 May 3;47(W1):W300–7.

44. Walsh I, Seno F, Tosatto SCE, Trovato A. PASTA 2.0: an improved server for protein aggregation prediction. Nucleic Acids Research. 2014 May 21;42(W1):W301–7.

45. Varadi M, De Baets G, Vranken WF, Tompa P, Pancsa R. AmyPro: a database of proteins with validated amyloidogenic regions. Nucleic Acids Research. 2017 Oct 13;46(D1):D387–92.

46. AmyPro database. https://amypro.net/#/entries/AP00047. Accessed 07 February 2021.

47. Jespersen MC, Peters B, Nielsen M, Marcatili P. BepiPred-2.0: improving sequence-based B-cell epitope prediction using conformational epitopes. Nucleic Acids Research. 2017 May 2;45(W1):W24–9.

48. Ponomarenko J, Bui H-H, Li W, Fusseder N, Bourne PE, Sette A, et al. ElliPro: a new structure-based tool for the prediction of antibody epitopes. BMC Bioinformatics. 2008 Dec;9(1).

49. Vita R, Mahajan S, Overton JA, Dhanda SK, Martini S, Cantrell JR, et al. The Immune Epitope Database (IEDB): 2018 update. Nucleic Acids Research. 2018 Oct 24;47(D1):D339–43.

50. Dunbar J, Krawczyk K, Leem J, Baker T, Fuchs A, Georges G, et al. SAbDab: the structural antibody database. Nucleic Acids Research. 2013 Nov 8;42(D1):D1140–6.

51. Altschul S. Gapped BLAST and PSI-BLAST: a new generation of protein database search programs. Nucleic Acids Research. 1997 Sep 1;25(17):3389–402.

52. Jack CR Jr, Bennett DA, Blennow K, Carrillo MC, Dunn B, Haeberlein SB, et al. NIA-AA Research Framework: Toward a biological definition of Alzheimer’s disease. Alzheimer’s & Dementia. 2018 Apr;14(4):535–62.

53. Hansson O. Biomarkers for neurodegenerative diseases. Nature Medicine. 2021 Jun;27(6):954–63.

54. Thijssen EH, La Joie R, Strom A, Fonseca C, Iaccarino L, Wolf A, et al. Plasma phosphorylated tau 217 and phosphorylated tau 181 as biomarkers in Alzheimer’s disease and frontotemporal lobar degeneration: a retrospective diagnostic performance study. The Lancet Neurology. 2021 Sep;20(9):739–52.

55. Popov KI, Makepeace KAT, Petrotchenko EV, Dokholyan NV, Borchers CH. Insight into the Structure of the

“Unstructured” Tau Protein. Structure. 2019 Nov;27(11):1710-1715.e4.
56. Luk C, Giovannoni G, Williams DR, Lees AJ, de Silva R. Development of a sensitive ELISA for quantification of three- and four-repeat tau isoforms in tauopathies. Journal of Neuroscience Methods. 2009 May;180(1):34–42.
57. Kapogiannis D, Mustapic M, Shardell MD, Berkowitz ST, Diehl TC, Spangler RD, et al. Association of Extracellular Vesicle Biomarkers With Alzheimer Disease in the Baltimore Longitudinal Study of Aging. JAMA Neurology. 2019 Nov 1;76(11):1340.
58. Jouanne M, Rault S, Voisin-Chiret A-S. Tau protein aggregation in Alzheimer’s disease: An attractive target for the development of novel therapeutic agents. European Journal of Medicinal Chemistry. 2017 Oct;139:153–67.
59. MAPT - Microtubule-associated protein tau - Homo sapiens (Human). UniProt. https://www.uniprot.org/uniprot/P10636. Accessed 07 February 2021.
60. MAPT protein (human) - STRING interaction network. STRING. https://version-11-5.string-db.org/cgi/network?networkId=bjUOQjbBGOix. Accessed 07 February 2021.
61.Gene-disease association data retrieved from DisGeNET v7.0. Integrative Biomedical Informatics Group GRIB/IMIM/UPF. DisGeNET. https://www.disgenet.org/browser/1/1/0/4137/. Accessed 07 February 2021.
62. MAPT protein expression summary. The Human Protein Atlas. https://v21.proteinatlas.org/ENSG00000186868-MAPT. Accessed 07 February 2021.
63. Gene summary - MAPT. Vesiclepedia. http://microvesicles.org/gene_summary?gene_id=4137. Accessed 07 February 2021.
64. Zhang W, Tarutani A, Newell KL, Murzin AG, Matsubara T, Falcon B, et al. Novel tau filament fold in corticobasal degeneration. Nature. 2020 Feb 12;580(7802):283–7.
65. Kadavath H, Jaremko M, Jaremko Ł, Biernat J, Mandelkow E, Zweckstetter M. Folding of the Tau Protein on Microtubules. Angewandte Chemie International Edition. 2015 Jun 19;54(35):10347–51.
66. Fontela YC, Kadavath H, Zweckstetter M. Structure of Tau(254-268) bound to F-actin. 2018 Mar 14; http://doi.org/10.2210/pdb5NVB/pdb.
67. Database APS. AlphaFold Protein Structure Database. https://alphafold.ebi.ac.uk/entry/P10636. Accessed 07 February 2021.
68. DisProt. https://disprot.org/DP01100. Accessed 07 February 2021.
69. MobiDB. https://mobidb.bio.unipd.it/P10636. Accessed 07 February 2021.
70. Tau (human). PhosphoSitePlus. https://www.phosphosite.org/proteinAction.action?id=1060&showAllSites=true. Accessed 07 February 2021.
71. iPTMnet Report P10636 MAPT. iPTMnet. https://research.bioinformatics.udel.edu/iptmnet/entry/P10636/. Accessed 07 February 2021.
72. InterPro. https://www.ebi.ac.uk/interpro/protein/UniProt/P10636/. Accessed 07 February 2021.
73. AmyPro database. https://amypro.net/#/entries/AP00012. Accessed 07 February 2021.
74. Suárez-Calvet M, Kleinberger G, Araque Caballero MÁ, Brendel M, Rominger A, Alcolea D, et al. sTREM 2 cerebrospinal fluid levels are a potential biomarker for microglia activity in early-stage Alzheimer’s disease and associate with neuronal injury markers. EMBO Molecular Medicine. 2016 Mar 3;8(5):466–76.
75. Rauchmann B-S, Schneider-Axmann T, Alexopoulos P, Perneczky R. CSF soluble TREM2 as a measure of immune response along the Alzheimer’s disease continuum. Neurobiology of Aging. 2019 Feb;74:182–90.
76. TREM2 - Triggering receptor expressed on myeloid cells 2 precursor - Homo sapiens (Human). UniProt. https://www.uniprot.org/uniprot/Q9NZC2. Accessed 07 February 2021.
77. TREM2 protein (human) - STRING interaction network. STRING. https://version-11-5.string-db.org/cgi/network?networkId=bRJP7ZiZpL76. Accessed 07 February 2021.
78. Gene-disease association data retrieved from DisGeNET v7.0. Integrative Biomedical Informatics Group GRIB/IMIM/UPF. DisGeNET. https://www.disgenet.org/browser/1/1/0/54209/. Accessed 07 February 2021.
79. TREM2 protein expression summary. The Human Protein Atlas.

https://v21.proteinatlas.org/ENSG00000095970-TREM2. Accessed 07 February 2021.

80. Gene summary - MAPT. Vesiclepedia. http://microvesicles.org/gene_summary?gene_id=54209. Accessed 07 February 2021.

81. Szykowska A, Chen Y, Smith TB, Preger C, Yang J, Qian D, et al. Selection and structural characterization of anti-TREM2 scFvs that reduce levels of shed ectodomain. Structure. 2021 Nov;29(11):1241-1252.e5.

82. Steiner A, Schlepckow K, Brunner B, Steiner H, Haass C, Hagn F. γ-Secretase cleavage of the Alzheimer risk factor TREM2 is determined by its intrinsic structural dynamics. The EMBO Journal. 2020 Aug 24;39(20).

83. Database APS. AlphaFold Protein Structure Database. https://alphafold.ebi.ac.uk/entry/Q9NZC2. Accessed 07 February 2021.

84. MobiDB. https://mobidb.bio.unipd.it/Q9NZC2. Accessed 07 February 2021.

85. TREM2 (human). PhosphoSitePlus. https://www.phosphosite.org/proteinAction?id=3100219&showAllSites=true. Accessed 07 February 2021.

86. iPTMnet Report Q9NZC2 TREM2. iPTMnet. https://research.bioinformatics.udel.edu/iptmnet/entry/Q9NZC2/. Accessed 07 February 2021.

87. InterPro. https://www.ebi.ac.uk/interpro/protein/UniProt/Q9NZC2/. Accessed 07 February 2021.
